# New putative phenol oxidase in ascidian blood cells

**DOI:** 10.1038/s41598-022-18283-9

**Published:** 2022-08-22

**Authors:** M. A. Daugavet, M. I. Dobrynina, T. G. Shaposhnikova, A. I. Solovyeva, A. G. Mittenberg, S. V. Shabelnikov, I. Yu. Babkina, A. V. Grinchenko, D. V. Ilyaskina, O. I. Podgornaya

**Affiliations:** 1 Institute of Cytology of Russian Academy of Sciences, St. Petersburg, Russia; 2 Saint-Petersburg State University, St. Petersburg, Russia; 3 Zoological Institute of Russian Academy of Sciences, St. Petersburg, Russia; 4 A.V. Zhirmunsky National Scientific Center of Marine Biology, Vladivostok, Russia; 5 Vrije Universiteit Amsterdam, 1081 HV Amsterdam, The Netherlands

**Keywords:** Evolution, Molecular biology, Zoology

## Abstract

The phenol oxidase system is ancient and ubiquitously distributed in all living organisms. In various groups it serves for the biosynthesis of pigments and neurotransmitters (dopamine), defence reactions and tissue hardening. Ascidians belong to subphylum Tunicata, which is considered the closest living relative to Vertebrates. Two phenol oxidases previously described for ascidians are vertebrate-like and arthropod-like phenol oxidases. In our present study, we described a new ascidian protein, *Tuphoxin,* with putative phenol oxidase function, which bears no sequence similarity with two enzymes described previously. The closest related proteins to Tuphoxin are mollusc haemocyanins. Unlike haemocyanins, which are oxygen transporting plasma proteins, Tuphoxin is synthesised in ascidian blood cells and secreted in the extracellular matrix of the tunic—ascidian outer coverings. Single mature transcript coding for this phenol oxidase can give several protein products of different sizes. Thus limited proteolysis of the initial protein is suggested. A unique feature of Tuphoxins and their homologues among Tunicata is the presence of thrombospondin first type repeats (TSP1) domain in their sequence which is supposed to provide interaction with extracellular matrix. The finding of TSP1 in the structure of phenol oxidases is new and we consider this to be an innovation of Tunicata evolutionary lineage.

## Introduction

Phenol oxidases (PO) comprise a non-homologous enzyme group that uses molecular oxygen for phenols’ oxidation. For example, the tyrosinase enzyme (EC 1.14.18.1) is a particular type of PO. Different organisms like molluscs^1,2^, arthropods^3,4^, or fungi^5,6^ use tyrosinase enzyme as a promoter of protein cross-linking^7^, although this enzyme is most commonly known for its role in pigmentation^8,9^. Tyrosinase and other PO are representatives of a large conservative group of copper-containing proteins (Table 1). The copper-containing protein superfamily is subdivided into three types depending on the amino acids coordinating copper in the active site^10–12^. For members of type I and type II copper atom is linked to histidine and cysteine or histidine and different N and/or O ligands, respectively. A pair of copper atoms, in the active site of tyrosinase, is coordinated by three histidines each, and this is a distinctive feature of type III copper proteins^13,14^. Other members of type III copper proteins are catechol oxidases and haemocyanins^15^. Copper cations in the active site bind oxygen molecule^16,17^ and use it for substrate oxidation^13,18^. Based on substrate preferences both tyrosinase and catechol oxidase meet the definition of “phenol oxidase”. The difference is that tyrosinase can oxidise mono-phenols or diphenols whereas catecoloxidase only diphenols^19^. Haemocyanins are also capable of enzymatic activity against phenols under special conditions^16,20,21^, but in the physiological state, they transport oxygen molecule without using it to actual oxidation of any substrate^22,23^.

**Table 1:** Classification of copper-containing proteins.

Copper-containing proteins	Type I "cupredoxins"	Plastocyanins Azurins Pseudoazurins Amicyanins Rusticyanins Cucumber basic proteins Stellacyanins^24^
	Type II	Amine oxidases Cu monooxygenases Nitrite reductase/multicopper oxidases CuZn superoxide dismutases^25^
	Type III	Haemocyanins^19,25^

Phenol oxidase reaction was documented in ascidian blood cells and it was restricted to morula cell type^26^. PO activity was also registered in the tunic—the extracellular structure of ascidian outer coverings^27,28^. Tunic PO activity could also be provided by morula cells^29,30^ that migrate from the blood vessels and degranulate in the tunic^31^. It was shown later that ascidian PO can also be activated during rejection reaction of genetically incompatible individuals of colonial species^32^ this process accompanied by degranulation of morula cells^33–35^. PO activity is registered at the site of injury or infection, thereby playing a role in immune response and wound healing^34,36–38^. Sequences of two phenol oxidases, similar to arthropod type PO, are described for ascidian *Ciona intestinalis* (CinPO 1, 2)^39^. They are activated by bacterial cell wall lipopolysaccharides (LPS) in blood cells supposedly of morula lineage, granular cells, and univacuolar refractile granulocytes^40,41^. CinPO-2 is also expressed in follicular cells and transported into the oocyte^42^. At the same time, vertebrate-like ascidian tyrosinase is expressed in the sensory pigment cells of the brain in the ascidian *Halocynthia roretzi*^43^. Yet no data is available concerning the role of two mentioned enzymes in the tunic formation.

Different blood cells can migrate through the epithelium of the vessels into the tunic^44–47^. For two ascidian species, *Styela rustica* and *Halocynthia aurantium* common features of tunic formation were described where morula cells secrete the components of the tunic matrix^28^. During the repair process in the site of injury of ascidian *S. rustica* morula blood cells are the most abundant among all cell types^31^. It was shown that morula cells of *S. rustica* contain two cell-type specific proteins with molecular weights of 48 kDa (p48) and 26 kDa (p26) visible as major bands at SDS-electrophoresis of morula cells proteins^31,48^. Polyclonal antibodies (AB) against p48 interacted with both major proteins of morula cells demonstrating their alikeness. AB also bind to the morula cells’ contents secreted into the tunic matrix^31^. Experiments on other ascidians from the order Stolidobranchia show that AB also bind to the morula cells and tunic matrix of *Styela coriacea, Boltenia echinata, Molgula* *citrina* and *H. aurantium* and test cells of reproductive system for all of them except *H. aurantium*^48^. In the present study, we described the gene, named *Tuphoxin* (Tunicate Phenol Oxidase), coding for p48 and several similar proteins in *S. rustica* and *H. aurantium*. We predicted the functional domains of these proteins and showed the phylogenetic relationships with proteins of other eukaryotes.

## Results

### Blood cell fractionation and immunostaining

**Figure 1:**
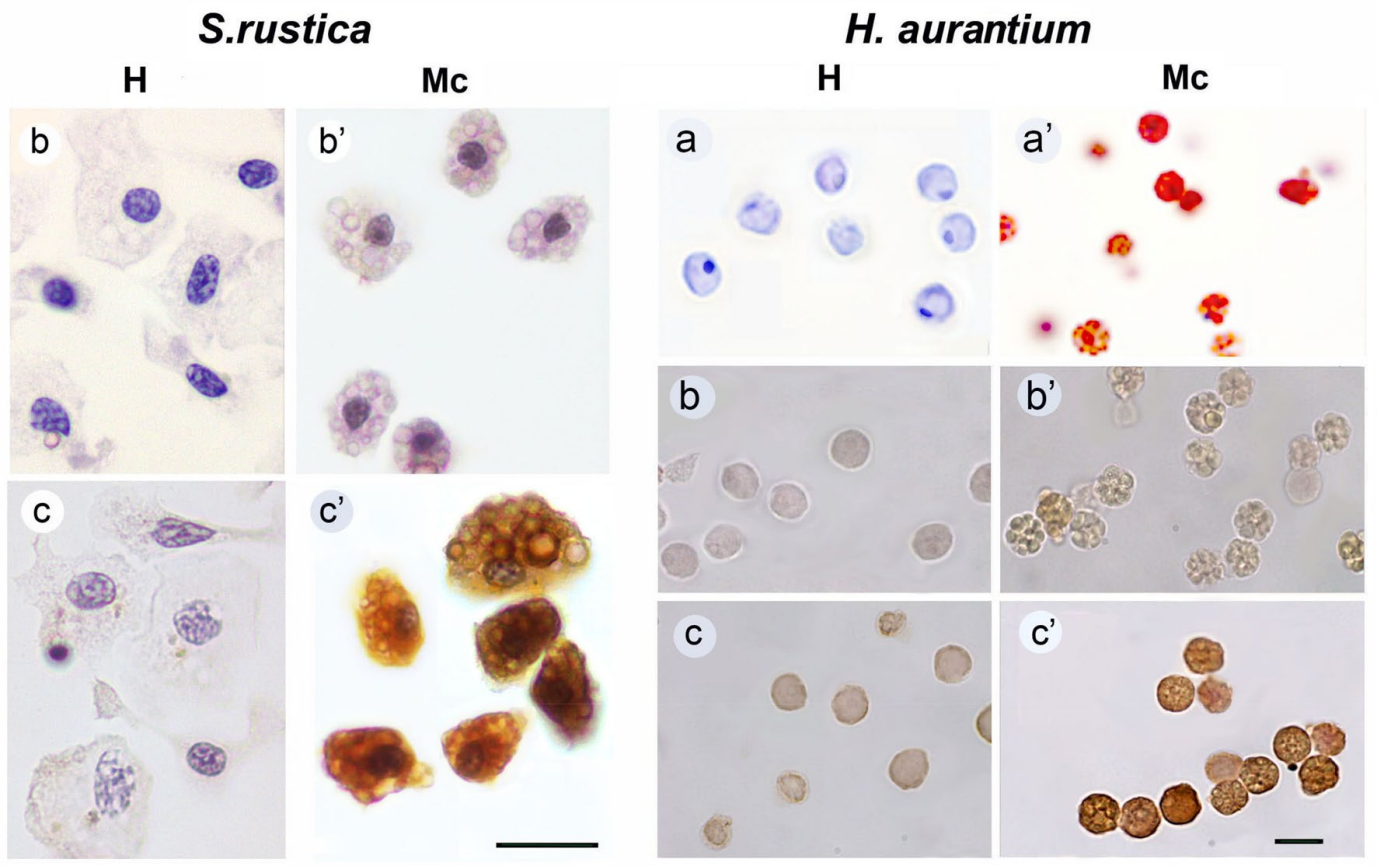
Cell fractions of hyalinocytes (H) and morula cells (Mc) of *S. rustica* and *H. aurantium.* (**a**, **a′**) *H. aurantium* cells stained with hematoxylin and eosin; (**b**, **b′**) *S. rustica* and *H. aurantium* cells, immunostaining without AB to p48 (control); (**c**, **c′**) *S. rustica* and *H. aurantium* cells, immunostaining with AB to p48. All *S. rustica* cells are additionally stained with hematoxylin. Scale bar—10 µm.

**Figure 2:**
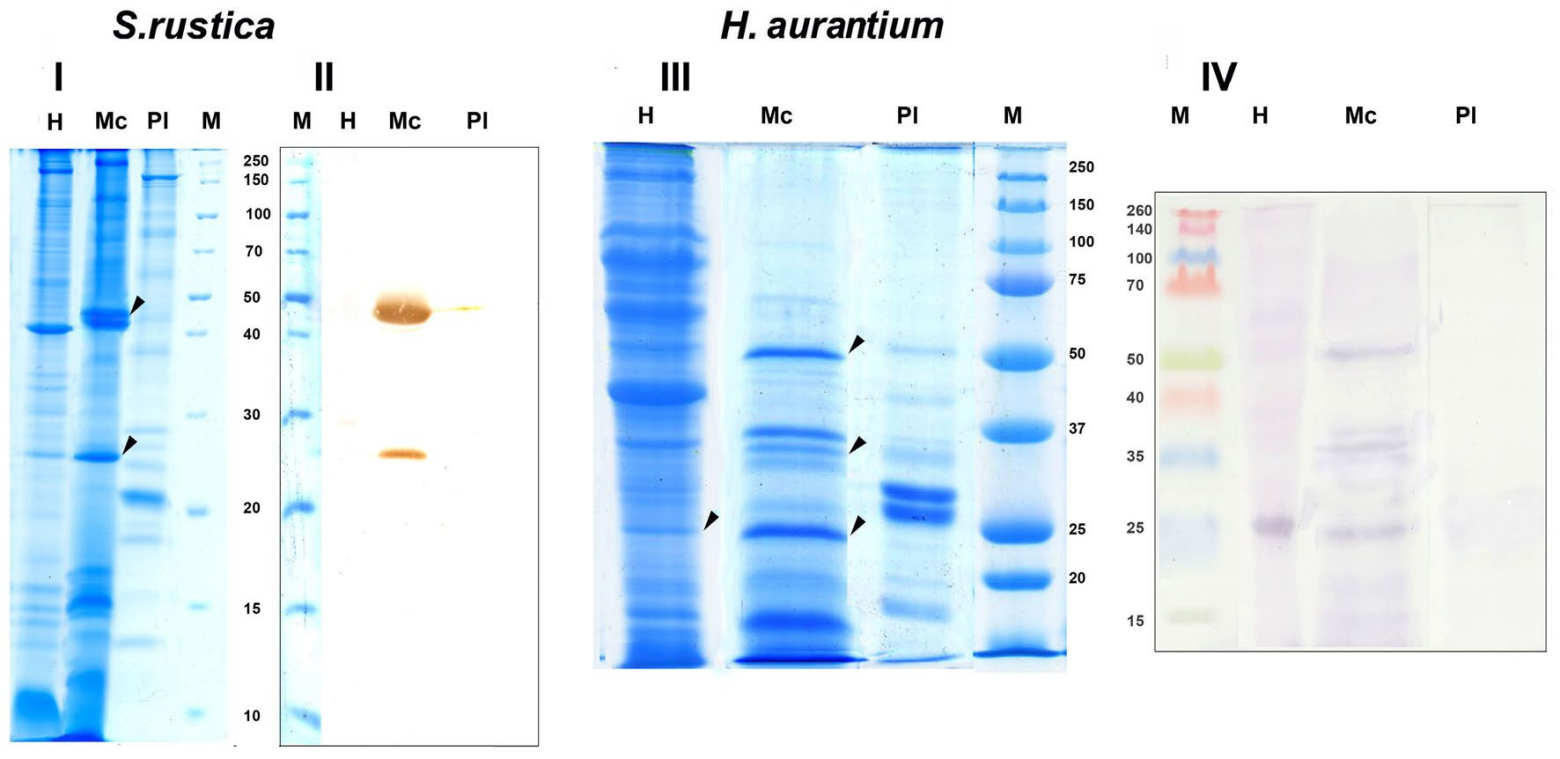
Electrophoresis and immunoblot of *S. rustica* and *H. aurantium* blood cell proteins*.* I, III—SDS-electrophoresis; II, IV—immunoblot. Hyalinocytes (H), morula cells (Mc), blood plasma (Pl), protein ladder (M). Protein ladder of panel II stained with amido-black. Arrows indicate protein bands immunoreactive to AB to p48, excised from the gel for MALDI analysis.

The composition of *S. rustica* cell fractions examined by phase-contrast microscopy was consistent with the results described previously^48,49^. In particular, fraction I enriched in hyaline amoebocytes, fraction II contained hyalinocytes and young morula cells, fraction III consisted of 95–97% of mature morula cells. The composition of *H. aurantium* cell fractions was similar to that of *S. rustica* and was additionally examined after hematoxylin and eosin staining. It showed that fraction I contained hyalinocytes (Fig. 1a) and fraction III contained mature morular cells (Fig. 1a′). Localisation of p48 or relative proteins in different types of blood cells was detected by immunohistochemical staining of cell fractions. AB raised against p48 of *S. rustica* interacted with *S. rustica* morula blood cells, while there was no specific interaction with hyalinocytes (Fig. 1, *S. rustica*, b, b′, c, c′). In blood cells of *H. aurantium* AB interacted with morula cells and also weak interaction with hyalinocytes was visible (Fig. 1, *H. aurantium*, b, b′, c, c′). In order to understand what proteins account for AB interaction, we performed an SDS-PAGE followed by a western blot. In morula cells of *S. rustica* AB bound with 48 kDa and 26 kDa bands (Ref.^48^ and present study Fig. 2, I, II). Morula cells of *H. aurantium* contain several major proteins with molecular masses of 48 and 26 kDa, and also three bands close to 35 kDa. All of them were immunoreactive (Fig. 2, III and IV, Mc). Hyalinocyte fraction of *H. aurantium* has no prominent protein bands except actin (Fig. 2, III, H), while immunostaining revealed a zone close to 26 kDa (Fig. 2, IV, H). Thus protein staining on immunoblot agree with the result of immunohistochemical staining of *H. aurantium* cell fractions. Immunoreactive proteins of *S. rustica* and *H. aurantium* were excised from the gel (Fig. 2, I, III, arrows) according to the Western blot staining for further mass spectrometric analysis.

### Sequence of *Tuphoxins*

Protein bands excised from the gel were digested by trypsin. The primary amino acid (aa) sequence of tryptic fragments was deduced by MALDI MS–MS and further used to find full-length sequences in protein databases. We used the translation of *de novo* sequenced draft transcriptome of blood cells belonging to *S. rustica* (BioProject ID:PRJNA772663) and whole body transcriptome available for *H. aurantium*^50^ as databases for proteins search. First, we determined sequences of short peptides belonging to 48 kDa and 26 kDa bands of *S. rustica* (Fig. 2, I, arrows). All the peptides from the two distinct protein bands belong to the same transcript from *S. rustica.* This transcript was cloned and sequenced (Supplementary Data 1). Based on BLAST search most of the hits have sequences identity < 40%, and those with bigger similarity are predicted proteins. Thus the transcript, and accordingly its gene was considered new and named *Styela rustica Tuphoxin* (Sru_*Tuph*).

**Figure 3:**
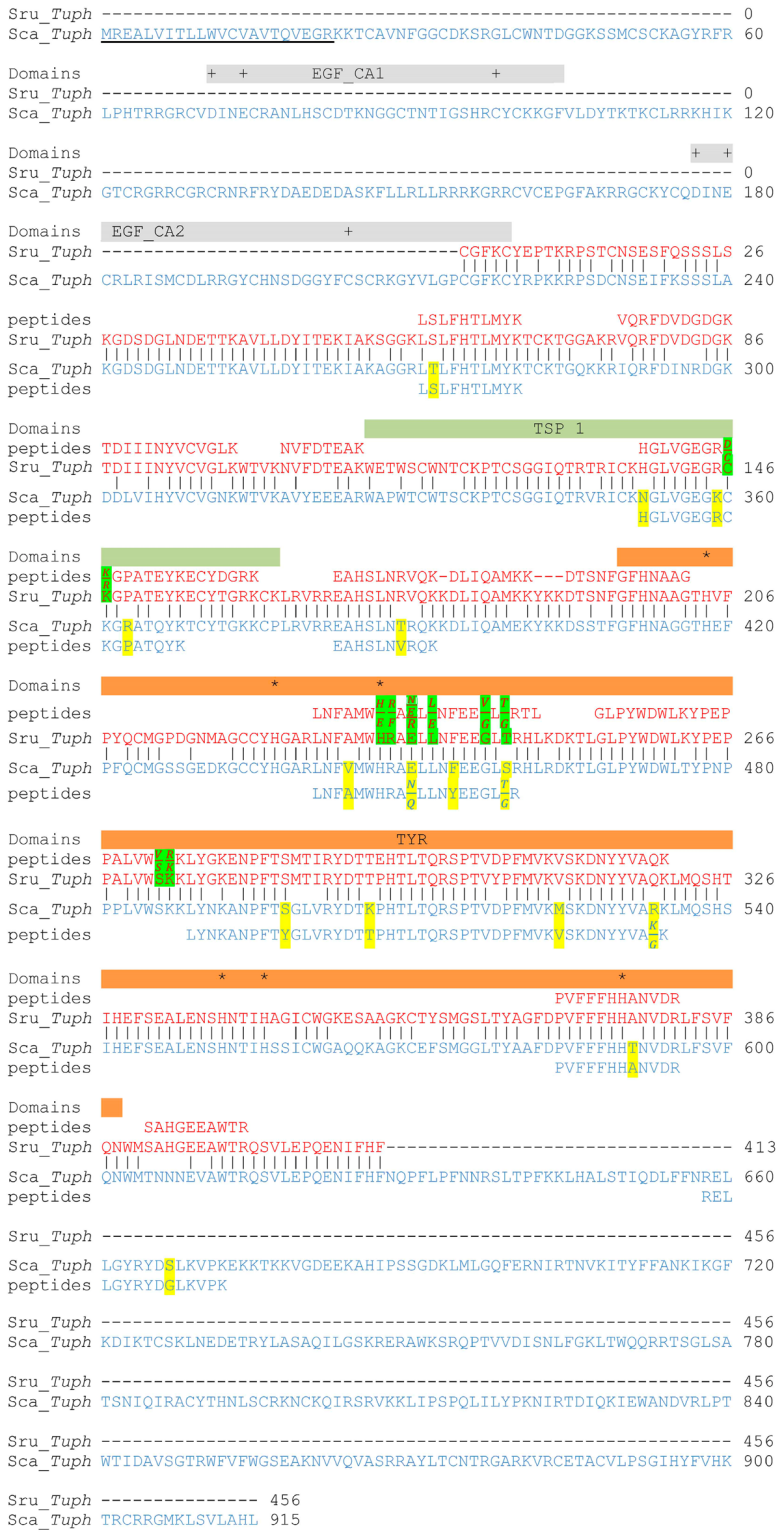
Alignment of tryptic peptides from 48 kDa protein band with *in silico* translated *Tuphoxin* transcripts. Transcripts of *S. rustica—*Sru_*Tuph* (red), and *S. canopus*—Sca_*Tuph* (blue). Vertical bars show aa that are identical for two species. Tryptic peptides are merged in longer sequences, with aa substitutions compared to translated transcripts highlighted in green (Sru_*Tuph*) and yellow (Sca_*Tuph*). The predicted signal peptide is underlined. Predicted conserved domains are marked by rectangles: grey (calcium-binding EGF-like—EGF_CA), green (thrombospondin first type repeat—TSP1), and orange (tyrosinase—TYR). Plus signs (+) indicate calcium-binding sites, the asterisks (*) indicate the active site amino acids.

We further used Sru_*Tuph* detected by mass spectrometry for sequence analysis. Tryptic fragments of 48 and 26 kDa bands possess several aa substitutions compared to in silico translated sequence (Fig. 3, Supplementary Fig. S1; substitutions highlighted green). The predicted Mr of the protein encoded by Sru_*Tuph* is 47 kDa. There is no stop-codon in the nucleotide sequence of the ORF, probably due to the incompleteness of transcriptomic data, so the real weight of the protein product could differ. In order to find the full ORF we used the whole body transcriptome of the close species *S. canopus*. The search based on peptides from 48 and 26 kDa bands again identified a single transcript of *S. canopus*—Sca_*Tuph* (Fig. 3, Supplementary Fig. S2). It contains a full predicted ORF that has start- and stop-codons. Several peptides identified by mass spectrometry have aa substitutions compared to the predicted aa sequence (highlighted yellow in Fig. 3, Supplementary Fig. S2). In addition, in three positions (Glu 450, Ser 459, Arg 533) peptides contain two variants of aa substitutions (highlighted yellow in Fig. 3, Supplementary Fig. S2). Calculated characteristics of mature protein are Mr of 103 kDa and pI 9.77. Thereby predicted molecular weight of protein product is greater than molecular weights of *S. rustica* protein variants based on SDS electrophoresis.

Tryptic peptides of *H. aurantium* immunoreactive proteins (Fig. 2, arrows) were also subjected to MALDI and their aa sequences were identified. All peptides derived from 48 kDa, 35 kDa and 26 kDa proteins of morula cells correspond to the same transcript named Hau_*Tuph1* (Supplementary Figs. S3, S4). On the other hand, the set of peptides of 26 kDa protein of morula cells may belong to different transcript Hau_*Tuph2* (Supplementary Fig. S5). Protein band detected in hyalinocytes correspond to Hau_*Tuph1* (Supplementary Fig. S6). The sequence of Hau_*Tuph1* starts with Gln (Q), so it may not correspond to full length ORF. Although the sequence of Hau_*Tuph2* starts with Met (M) it is shortened in comparison with Hau_*Tuph1*. The predicted Mr of protein products are 85 kDa for Hau_*Tuph1* and 63 kDa for Hau_*Tuph2*, which is greater than apparent masses of protein products based on SDS-electrophoresis. Moreover for both *S. rustica* and *H. aurantium* there were more immunoreactive protein bands in the gel than unique transcripts detected by MALDI (Table 2), which may indicate posttranslational proteolytic processing.

**Table. 2:** Protein products encoded by *Tuphoxin* of *S. rustica* and *H. aurantium.*

Ascidian species	*Styela rustica*	*Halocynthia aurantium*		
Transcript	Sru_*Thuph*	Hau_*Tuph1*		Hau_*Tuph2*
Database ID	Merged: NODE_28777, NODE_67444	Haaura.CG.MT P2014.S1292 g08050.02.p		Haaura.CG.MTP2014.S1292 g08050.01.p
Predicted Mr	47 kDa	85 kDa		63 kDa
Immunoreactive bands (Mr)	48 kDa 26 kDa	48 kD 35 kDa 26 kDa	26 kDa	26 kDa
Cell type	Morula cells	Morula cells	Hyalinocytes	Morula cells

### Domain composition of predicted proteins

Further we analysed conservative functional domains that can exist in the predicted proteins of *S. rustica, S. canopus* and *H. aurantium*. The longest transcript found belongs to *S. canopus.* Translation of Sca_*Tuph* contains N-terminal signal peptide (Fig. 3), and thus it was considered to encode a secretory protein. Four functional domains were also predicted: two calcium-binding EGF-like domains (EGF_CA1 and EGF_CA2—pfam07645, smart00179 respectively), thrombospondin first type repeat (TSP1—smart00209), and tyrosinase (Tyr—pfam00264) domain. EGF-like domains contain conserved amino acids required for calcium binding (plus signs in Fig. 3). Four functional domains are found in full sequence, nevertheless, peptides determined by mass spectrometry in 48 kDa band fall into the region corresponding to the last two domains, thrombospondin, and tyrosinase (Fig. 3). Peptides from the band of 26 kDa are situated only in the predicted tyrosinase domain (Supplementary Fig. S2). The same peptide distribution is observed for Sru_*Tuph* protein products (Fig. 3; Supplementary Fig. S1).

*H. aurantium* transcript Hau_*Tuph1* encodes 48 kDa and 35 kDa proteins of morula cells and 26 kDa protein of hyalinocytes. In the translation of Hau_*Tuph1* no signal peptide was found. The search for conserved functional domains identified thrombospondin first type repeat (TSP1—smart00209), tyrosinase (Tyr—pfam00264), and domain of cupredoxin family (CuOx—cl19115) (small copper-containing blue proteins, see Table 1) (Supplementary Figs. S3, S4, S6). The second transcript Hau_*Tuph2* includes peptides found in 26 kDa protein band of morula cells. Hau_*Tuph1* and Hau_*Tuph2* are 100% identical apart from the fact that Hau_*Tuph2* is shortened*.* It lost part of N-terminal sequence. Moreover, it has a gap in the middle so that it lacks the TSP1 domain (Supplementary Fig. S5), thus including only the tyrosinase domain (pfam00264) and the domain of the cupredoxin family (cl19115).

**Figure 4:**
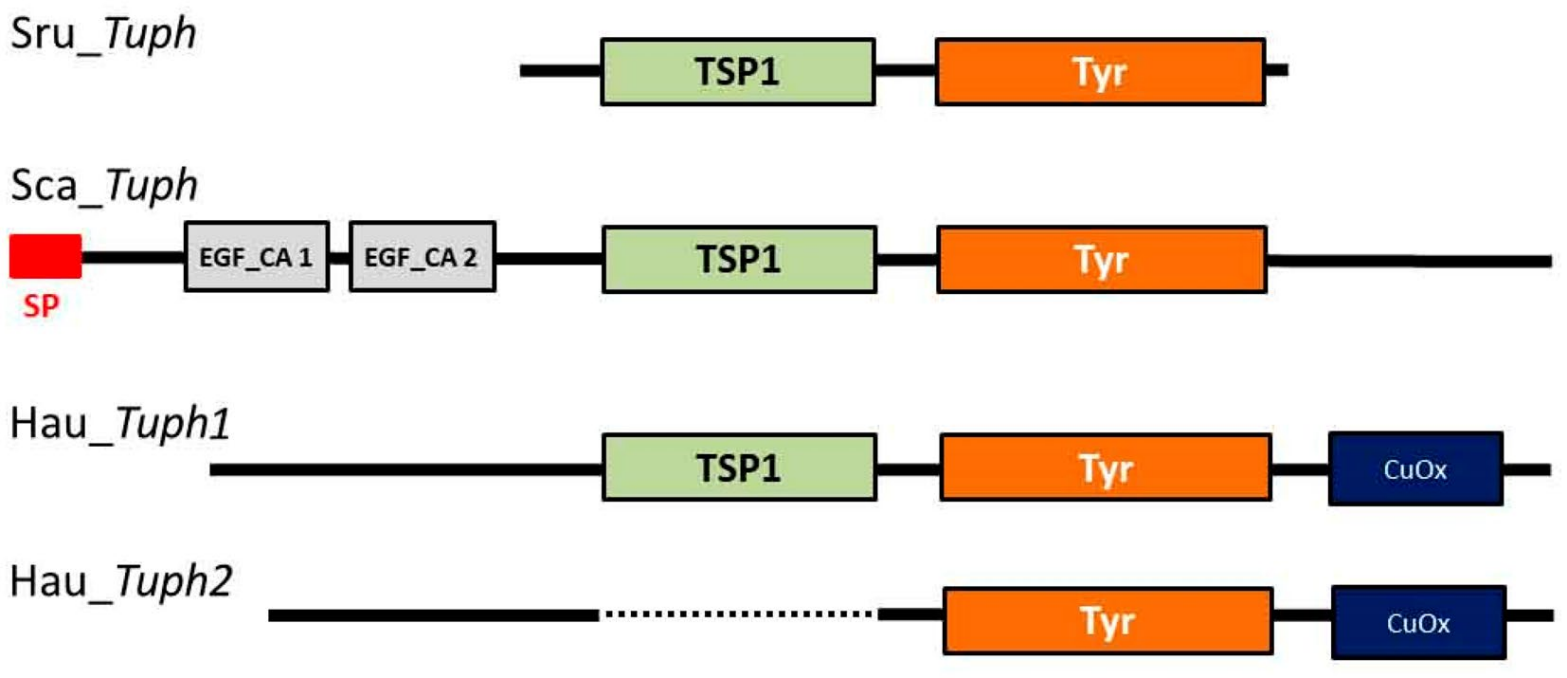
Diagram of domain composition based on the alignment of four predicted amino acid sequences encoded by *Tuphoxins* of *S. rustica* (Sru_*Tuph*)*, S. canopus* (Sca_*Tuph*) and *H. aurantium* (Hau_*Tuph1,2*). *SP* Signal peptide, *EGF_CA* epidermal growth factor calcium-binding domain, *TSP1* thrombospondin first type repeat, *Tyr* tyrosinase, *CuOx* domain of cupredoxin family.

Summing up we conclude that four different functional domains can constitute protein products of *Tuphoxins* investigated in the present study. Based on the alignment (Supplementary Fig. S7) of deduced amino acid sequences and positions of functional domains we built a comparison diagram (Fig. 4). A common feature for all proteins is the presence of the tyrosinase domain, which is probably recognised by the AB used initially.

### Functional predictions

**Figure 5:**
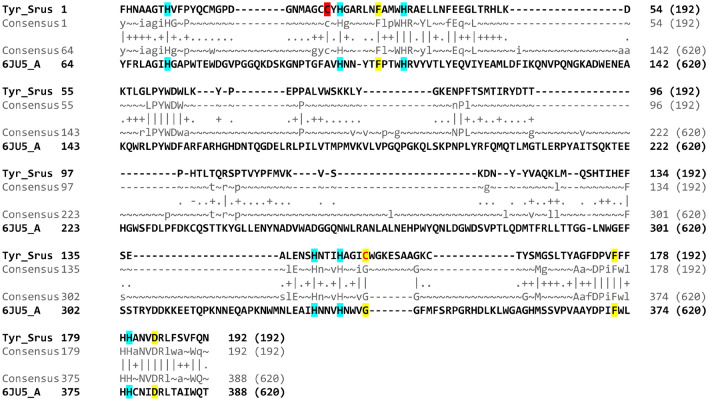
Results of HHpred search. Pairwise alignment and alignment of consensus sequences for tyrosinase domain of *S. rustica Tuphoxin* (Tyr_Srus) and *Aspergillus oryzae* (6JU5_A) tyrosinase region between 64 and 388 amino acids. Conservative histidines binding the Cu-ion highlighted in blue, amino acids specific for alfa tyrosinases^19^ highlighted in yellow with substitution typed red, cysteine forming the covalent linkage with histidine^51^ highlighted in red.

Potential enzymatic activity of the tyrosinase domain can be assessed based on the structure of the active site. The best match in the database of protein structures (PDB) was observed for Sru_*Tuph* tyrosinase domain with fungi *Aspergillus oryzae* tyrosinase (6JU5_A). Based on the alignment we confirmed the presence of copper-binding histidines (Fig. 5, highlighted blue; Fig. 3—asterisks) required for active site formation^51^. Cysteine residue forming an unusual covalent linkage with histidine^51^ is also present (Fig. 5, highlighted red). Sru_*Tuph* tyrosinase domain possesses conservative Phe and Asp next to metal-binding histidines characteristic for alfa tyrosinase subtype^19^. On the other hand, it has Gly replaced to Cys next to histidine in the copper-binding site B (Fig. 5, red highlighted yellow). This substitution is also present in several other alfa tyrosinases^19^. According to the paper mentioned all secreted tyrosinases belong to the alfa subtype. In agreement with this full-length protein product of Sca_*Tuph* contains signal peptide probably targeting the protein to secretion. On the basis of the results obtained we propose that protein products of *Tuphoxins* belong to the alfa subtype of tyrosinases. Even though we can detect sequence similarity with fungi tyrosinase, bioinformatic predictions can’t reliably distinguish between tyrosinase and catechol oxidase (Table 1). Thus we propose that *Tuphoxins* protein products are related to “phenol oxidases”.

TSP1 repeat, which is also present in longer protein products of *Tuphoxins*, has various functions in the extracellular compartment^52^. We searched for similarity with previously annotated proteins in UniProtKB/Swiss-Prot database. TSP1 repeat of Sru_*Tuph* showed maximum similarity with Human adhesion G protein-coupled receptor (O60242). TSP1 repeat of Sca_*Tuph* shows maximum similarity with the TSP domain of *Caenorhabditis briggsae* zinc metalloprotease Nas-36 (Q61EX6), indispensable for moulting process^53^. Alignments are shown in Supplementary Fig. S8. No reliable similarity was found for Hau_*Tuph*.

### Phylogeny of tyrosinase domain

**Figure 6:**
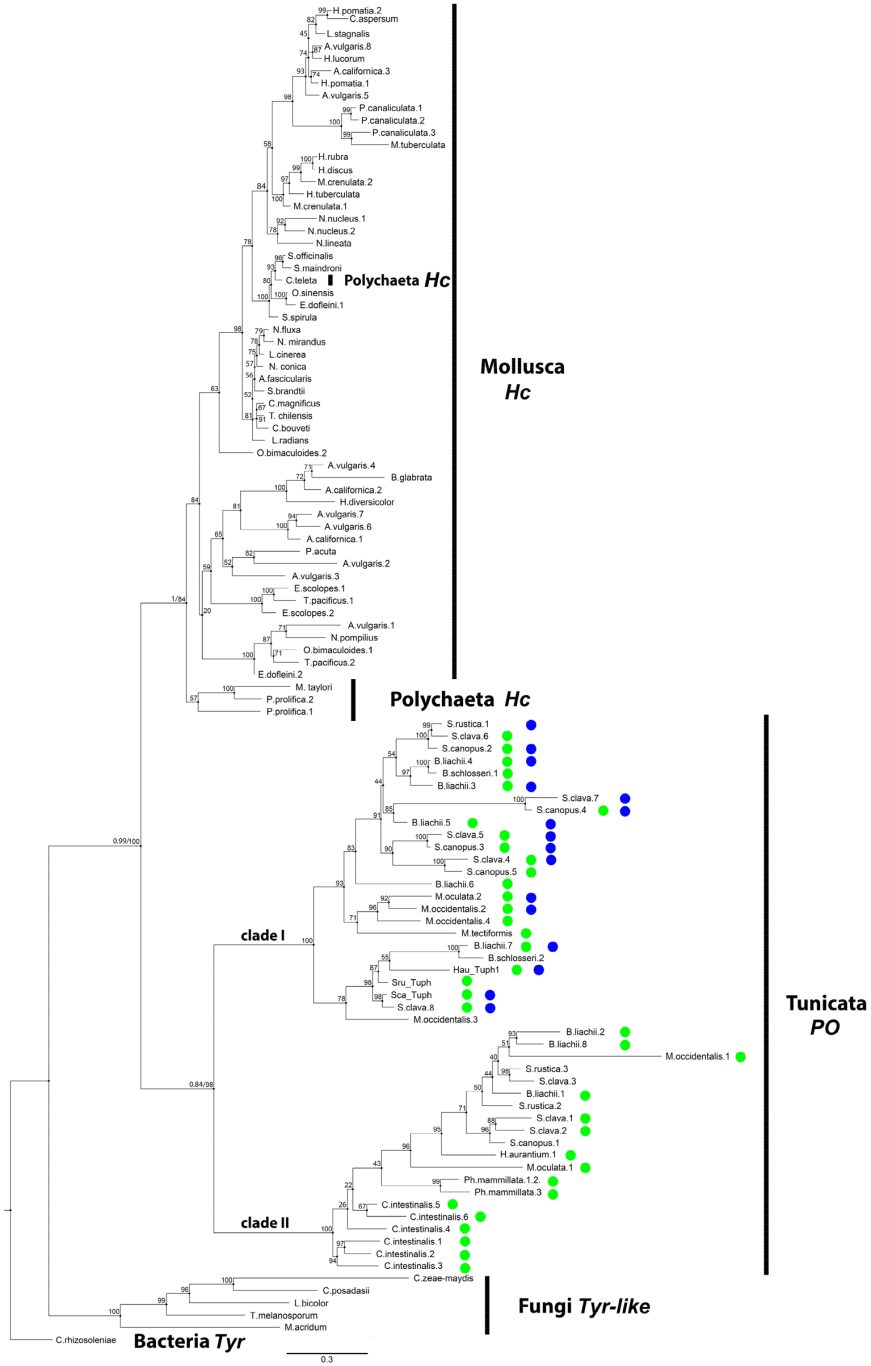
Phylogenetic analysis of tyrosinase domains of *Tuphoxin* homologues. A representative phylogenetic tree based on maximum likelihood (ML) inference. *Hc* haemocyanin, *PO* phenol oxidase, *Tyr* tyrosinase. Presence of cupedoxin-like sequence (blue cycles) and thrombospondin first type repeat-TSP1 domain (green cycles) is indicated for Tunicata POs. Statistical support is indicated at the nodes; for main branches first number is Bayesian Inference (BI) posterior probabilities; second number is ML bootstrap support. For all other branches only bootstrap support is indicated. Accession numbers of the sequences used in this tree can be found in Supplementary Table S1.

Using the isolated sequence of Sca_*Tuph* tyrosinase domain as a query we found homologues sequences belonging to Bacteria, Fungi, Annelida, Mollusca, and Tunicata. All sequences with reliable similarity were filtered to 90% identity to get rid of redundant data and then made up a dataset of 110 sequences (Supplementary Table S1). This dataset was used to construct a phylogenetic tree by ML (Fig. 6) and Bayesian (Supplementary Fig. S9) methods. Figure 6 shows phylogenetic relations inferred by ML with indicated bootstrap values for all branches, and posterior probabilities from the Bayesian method indicated for main branches. Our tree topology is consistent with the tree topology of alfa tyrosinases previously published^19^. Other POs of Tunicates described previously are arthropod-like sequences and belong to beta- subtype tyrosinase (Supplementary Table S2). We can’t find any reliable similarity with those proteins using BLAST search. Hence we included only the most conservative amino acid regions of those PO in alignment (Supplementary Data 2, BLOCKs) and constructed phylogenetic tree by Bayesian method. All arthropod-like POs clustered in separate clade occupying more basal position than Mollusc, Polychaeta and Tunicata alfa-tyrosinases (Supplementary Fig. S10, clade III).

Thus *Tuphoxins* and homologues sequences in Tunicata are close to mollusc haemocyanins, and both of these protein groups have a common protein predecessor with fungi alfa tyrosinases. Two well supported clades among sequences belonging to tunicates are visible. Notably, eight out of 11 studied species have alleles in both of these clades (Fig. 6, Supplementary Table S3). Hence we consider that duplication event of *Tuphoxin* ancestor gene took place in Tunicata clade.

Although the sequence of tyrosinase domain was used to resolve phylogenetic relations we can mark presence or absence of other domains in the full-length sequences used in phylogeny construction. TSP1 domain in junction with tyrosinase domain is present only in sequences belonging to tunicates (Fig. 6, green cycles). Cupredoxin domain is hard to be predicted but nevertheless, cupredoxin-like sequences are visible on alignment (Supplementary Figure S11). Presence of cupredoxin-like sequence is marked on ML tree by blue cycles (Fig. 6, Supplementary Table S4), such sequences are concentrated in one of the two tunicate clades. Cupredoxin domain is also part of mollusc haemocyanins^54^. Thus we may conclude that cupredoxin-like sequence was inherited from a common ancestor of mollusc and tunicate alfa tyrosinase, while the presence of TSP1 in junction with alfa tyrosinase is tunicate innovation.

## Discussion

### Protein products of *Tuphoxin*

**Figure 7:**
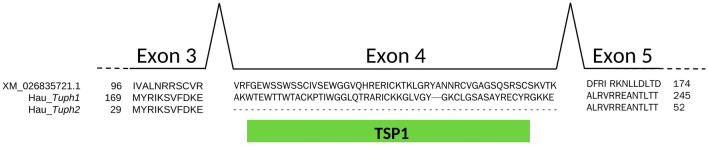
Alignment of *C. intestinalis* hemocyanin (ncbi ID XM_026835721.1) with translations of two *H. aurantium Tuphoxins*: Hau_*Tuph1* and Hau_*Tuph2*. Intron positions in chromosome DNA of *C. intestinalis* is indicated by arches. Position of thrombospondin first type repeat (TSP1) domain is shown by green rectangle.

In this study, we describe a new protein, synthesised in ascidian blood cells. The gene coding for this protein was named *Tuphoxin* after Tunicate Phenol Oxidase. In two ascidian species studied *S. rustica* and *H. aurantium* one unique transcript of *Tuphoxin* was found for each species though giving multiple protein products. Two transcripts Hau*_Tuph1* and Hau*_Tuph2* of *H. aurantium* are identical in their sequence but differ in a short gap in the middle of the sequence. Genome information about *H. aurantium* is available (GCA_013436065.1), but we can’t find *Tuphoxins* in the genome assembly. Thus intron–exon structure was assessed on the similar sequence of ascidian *C. intestinalis* (XM_026835721.1). It shows that the fourth exon is encoding the TSP1 domain and the position of this exon correspond to the gap in the Hau_*Tuph2* sequence (Fig. 7). Based on this notion we may assume that Hau_*Tuph1* and Hau_*Tuph2* resulted from alternative splicing.

The shorter transcript of *H. aurantium* must produce a protein with the predicted Mr of 63 kDa, which is greater than Mr based on SDS-electrophoresis (48 kDa). Thus another possible explanation is limited proteolysis of the nascent protein. It was shown previously that arthropod beta subtype PO exists in the form of proenzyme and is activated through limited proteolysis^55,56^. It is also true for the alfa subtype tyrosinase present in plants^57^. In echinoderms^58,^ cephalochordates^59^ and tunicates^58,60,61^ limited proteolysis of PO in vitro increased significantly its activity, but it is also active without proteolytic activation or due to spontaneous activation. According to our data predicted molecular mass based on the full transcript of *S. canopus* is 103 kDa. Protein products identified on SDS-electrophoresis with Mr ranging from 48 to 26 kDa, may represent different truncated forms of a single precursor molecule. MALDI determined peptides of 48 kDa zone of *S. rustica* lay in the central region of the sequence. Thus peptides positions may show the real borders of the mature protein. It is possible that high molecular weight forms exist but are very rare; they could be detected after immunoprecipitation of morula cells lysate with antibodies to 48 kDa protein. It is worth mentioning, that arthropod ProPO are supposed to be truncated after release in plasma. In case of *Tuphoxin* protein products, they were isolated from blood cells before their release, thus they must be truncated being inside the cell.

We found *Tuphoxin* protein products associated with two types of blood cells. These were morula cells of ascidian *S. rustica* and hyalinocytes and morula cells of ascidian *H. aurantium*. Previous data suggest that PO reaction is restricted to morula cells^26^ or their analogues cell types: granulocytes and unilocular refractile granulocytes in *Ciona inestinalis*, compartment cells in *Phallusia mammillata*^61^. In our study hyalinocytes of *H. aurantium* interacted with AB against tuphoxin but this labeling was week and concentrated on the cell surface. Western blot and masspectrometry detected a short protein product (26 kDa) of tuphoxin in those cells. Hence it is probable that tuphoxin from degranulated morula cells is bound to hyalinocytes surface. Knowing morula cells to degranulate in the tunic matrix we assume *Tuphoxin* protein products to be the components of the tunic ECM. On the other hand, to the best of our knowledge hyalinocytes remain in circulation and don’t enter tunic matrix.

### Functional characteristic

Four different functional domains can be predicted in *Tuphoxin* protein products. Those are EGF-like, TSP1, tyrosinase, and cupredoxin-like. EGF-like domains were described previously for different components of PO system in molluscs^62^, insects^63^, and ascidians^43^. In insect *Holotrichia diomphalia* EGF-like domains work as pattern recognition molecule binding bacterial lipopolysaccharides and are supposed to activate PO system^63^. Ca-binding EGF-like domains (PF07645) were predicted in Sca_*Tuph* sequence based on in silico translation, but no peptides were found by the MALDI approach corresponding to this part of the sequence. It is possible that those domains are cut away at some early stage of protein processing.

Other predicted conservative domains include peptides detected by MALDI. According to the positions of those peptides, 48 kDa protein product of Sru_*Tuph* contains two functional domains, thrombospondin, and tyrosinase. Peptides of the shorter protein product which is 26 kDa fall only to the tyrosinase domain. This protein most likely contains only one functional domain—tyrosinase. This domain is also present in all protein products of Sca_*Tuph* and Hau_*Tuph1,2*. Thus tyrosinase domain was considered a mandatory part for all protein products of *Tuphoxins*. Nevertheless, the presence of the tyrosinase domain doesn’t necessarily evidence for tyrosinase activity, so we would rather speak of *Tuphoxin* protein product as related to “phenol oxidases”. PO enzymes modify various phenolic substrates producing highly reactive molecules like quinones or their derivatives^13^. Those molecules further react with amino acid side chains resulting in crosslinking of proteins, a procedure known as sclerotisation or phenolic tanning. For instance, quinones can act as cross-linking molecules for wound healing^64,65^. They also polymerise to form melanin capsules around parasites^66,67^ or directly kill microbial pathogens^68^. Moreover, the process of sclerotisation takes place in various structures of invertebrates: mussel byssus^69,70^, insect cuticles^71–73^ and squid beaks^1,74^, where it serves as a mechanism of tissue hardening. Knowing that morula cells degranulate in the tunic of ascidians^49^ we suppose that the tyrosinase domain in the structure of *Tuphoxin* protein products is involved in tunic sclerotisation.

Alongside with tyrosinase domain, longer protein products contain the thrombospondin domain. The predicted structure is a short repeat segment characteristic of thrombospondins and belongs to 1-th type repeats (TSP1). Thrombospodins are multimeric Ca-binding glycoproteins acting at the cell surfaces and in the extracellular matrix and referred to as “fundamental components of the extracellular interaction systems of metazoan” (p 2187 in 75). TSP1 is responsible for cell adhesion^75^, migration, and support of cell shape^76^. The role of TSP domain in ECM formation is also demonstrated by its involvement in the molting process in nematodes^53^, and we show sequence similarity of Sca_*Tuph* TSP1 to nematode *Caenorhabditis briggsae* protein. Proteins with TSP1 were described previously for ascidian *Ciona intestinalis*^77,78^. The novelty of our study was to find TSP1 as a part of PO related enzyme. We assume that the presence of TSP1 domain may lead to the interaction of *Tuphoxin* protein products with other components of ECM thus participating in tunic construction.

MALDI peptides of *H. aurantium* proteins show the existence of the fourth functional domain in the structure of Hau_*Tuph1,2* protein products. This is cupredoxin, belonging to I type copper containing proteins. The functional association of cupredoxin and tyrosinase domains could be quite ancient. In bacteria of genus *Streptomyces* cupredoxin-like protein MelC1 is coexpressed with tyrosinase and proposed to be involved in copper binding and loading it into the active site of the enzyme^79^. Though in the case of ascidian cupredoxin domains no histidines or cysteines were present in the positions essential for copper binding. There are other described cases of loss of the ability to copper binding by cupredoxin domains, for example in haemocyanin of mollusc *Megathura crenulata* or ephrin ectodomain of mouse^80,81^. In mollusc haemocyanins cupredoxin domain may serve for the assembly of functional units^54^ that is usual for multimeric haemocyanin complexes^17^. Possible dimerisation after LPS inoculation was also observed in *C. intestinalis* for CinPO1^26,61^. That enzyme belongs to the beta subtype tyrosinase. Though it is possible that cupredoxin-like sequence in *Tuphoxin* protein products, belonging to alfa tyrosinases, might be also involved in oligomerisation.

### Ancestry and evolution

Despite the fact that ascidian tyrosinases were described previously: arthropod-like tyrosinase^40^, vertebrate-like tyrosinase, and tyrosinase-related proteins^43^, *Tuphoxin* protein products have very low sequence similarity to those proteins. Wherein relative sequences that were found by BLAST belong to other Ascidiacea as well as to Molluscks, Annelida, Fungi, and Bacteria. We didn’t meet other tunicates, Thaliacea and Appendicularea, during our search. It may be due to the secondary loss of *Tuphoxin* related genes because their coverings are soft and transparent^82,83^ probably with no sclerotisation. Thus tyrosinase domain of *Tuphoxins* has common ancestors among metazoans with mollusc and annelida. We may also argue that *Tuphoxin* encoded tyrosinase domains preserve ancient features in their sequences since BLAST algorithm finds their reliable similarity with bacterial protein. All tyrosinases are divided into three subtypes: alfa, beta and gamma^19^. The most ancient among them are alfa, which genes are present in bacterial genomes. They are secreted proteins, while others are cytosol or membrane bound enzymes. In perfect agreement with this, the product of Sca_*Tuph* is predicted to be a secreted protein. All *Tuphoxin* tyrosinase domains cluster at the phylogenetic tree with mollusc haemocyanins, which also belong to the alfa subtype. Moreover, *Tuphoxin* protein products preserve essential amino acids in the active site specific for alfa subtype tyrosinases.

The presence of other functional domains apart from tyrosinase in full sequences was assessed and overlaid on the phylogenetic tree. Cupredoxin domains are recognised only in Hau_*Tuph1,2* protein products, but similarity at sequence level on our own alignment of ascidian proteins may show the presence of cupredoxin-like regions in multiple homologues. Two clades of sequences are visible with high bootstrap support in Tunicata branch. Those two clades don’t correspond to any taxonomic groups inside subphylum Tunicata, on the contrary, most species have sequences in both of these two clades. This topology may indicate an ancient duplication event of *Tuphoxin* ancestor sequence. Cupredoxin-like sequences are present in one of the clades and are absent in another clade (Fig. 6). According to literature data the cupredoxin domain is also present in molluscs haemocyanins^54,83,84^. Thereby ascidian cupredoxin in the structure of alfa tyrosinases may be inherited from common ancestor protein with haemocyanins but lost in one of the alleles after the duplication event.

**Figure 8:**
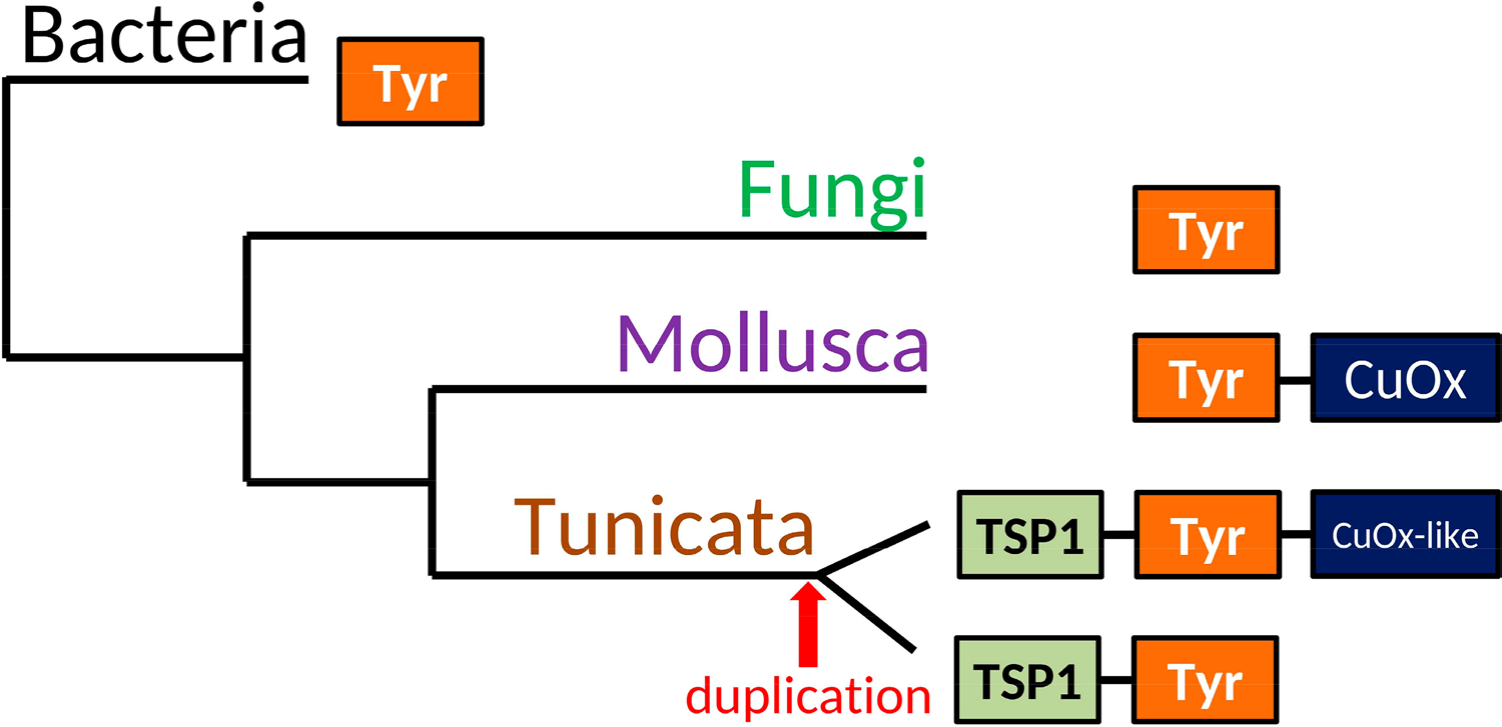
Schematic representation of domain composition in alfa tyrosinases belonging to different taxonomic groups. *TSP1* thrombospondin first type repeat, *Tyr* tyrosinase, *CuOx* domain of cupredoxin family.

Unlike the cupredoxin-like sequence TSP domain in alfa tyrosinases is limited to the tunicate branch. Schematic representation of phylogenetic groups and domain composition of their alfa tyrosinases is presented at Fig. 8. Both tyrosinase and TSP1 are widely distributed in eukaryotes^85–87^, but proteins containing simultaneously those two domains are currently found only for tunicates. As we know from described functions of TSP domain^86^ its appearance may indicate a functional connection to ECM and based on our findings TSP domain of *Tuphoxin* protein product may connect to ECM of the tunic.

## Conclusions

In the present study we describe *Tuphoxin*—a new protein of ascidians related to phenol oxidases. We consider it to participate in the tunic formation by means of two functional domains: alfa subtype tyrosinase domain which could fulfill enzymatic function and TSP1 domain which may interact with ECM components. The tunic is a unique extracellular structure and functional adaptation of Tunicata^88,89^. We may propose that prerequisite for the tunic construction was appearance of TSP1 in conjunction with alfa tyrosinase domain. Moreover, for the first time, we demonstrate TSP1 repeats in type III copper proteins and suppose this to be an innovation of tunicate evolutionary lineage.

## Methods

### Animals

We used ascidians of two species *Styela rustica* (Styelidae) and *Halocynthia aurantium* (Pyuridae) in our study. Ascidians *S. rustica* were collected around Fettakh Island near the Biological Station of the Zoological Institute of the Russian Academy of Sciences at Cape Kartesh (Kandalaksha Bay, the White Sea) in June–August of 2018–2021. Ascidians were collected either in the sublittoral zone (depth up to 10 m), or from artificial substrates (depth 3 m). Before the experiment animals were kept in cages at 5–7 m depth below the water surface. The required number of ascidians was taken from cages for blood sampling; the animals were kept for a short time in aquariums with aerated seawater in isothermal room (at 10 °C). Ascidians *H. aurantium* from the Sea of Japan were collected in the sublittoral zone near the MBS IBM RAS "Vostok" in November 2018. Before the blood sampling, ascidians were kept in aquariums at 6 °C and returned to their natural environment after the experiment.

### Blood collection

The manipulations were carried out at 10 °C.The tunic was cleaned of epiphytes, washed thoroughly, and dried with absorbent paper. The sampling area was sterilized with 70% ethanol and the tunic was incised with a razor blade to the muscular layer without injuring the internal organs. The blood exuding from the incision was collected by pipetting and mixed with anticoagulant solution 1:1 (AS: 0.3 M NaCl, 20 mM KCl, 15 mM EDTA, 10 mM HEPES pH 7.6 for *S. rustica*^31^; AS: 435 mM NaCl, 10.7 mM KCl, 27 mM Na_2_SO_4_, 16.6 mM C_6_H_12_O_6_; 12 mM HEPES, 5 mM EDTA for *H.aurantium* (modified according to Ref.^90^. The obtained cells were separated in a stepwise Percoll gradient.

### Preparation of blood cell fractions

**Figure 9:**
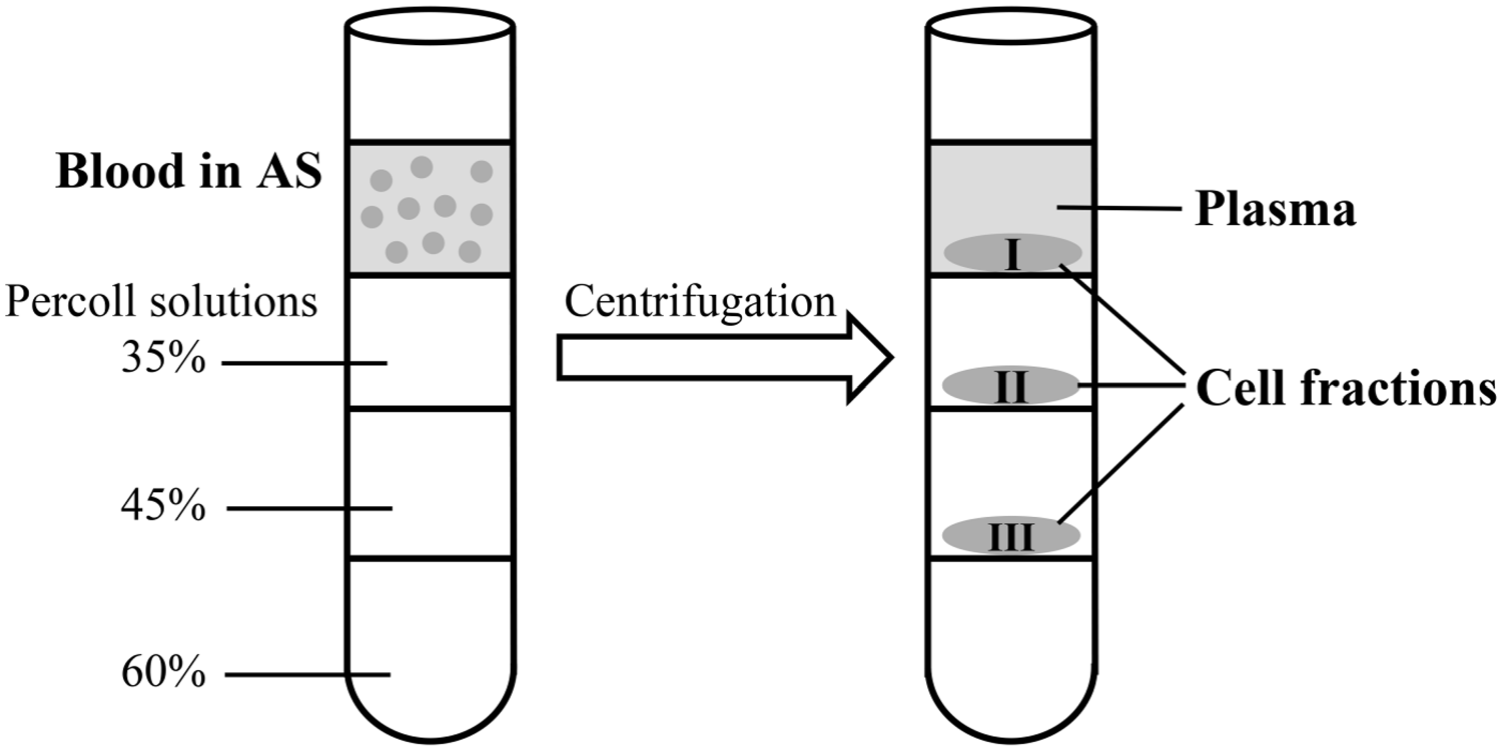
Scheme of blood cells fractionation in Percoll density gradient. *AS* anticoagulation solution.

Percoll solution (Sigma) was mixed with appropriate volumes of AS to obtain final concentrations of 60, 45, and 35%. Two millilitres of each mixture was overlaid sequentially into a glass centrifuge tube. Three millilitres of blood sample mixed with AS (1:1) were layered onto the Percoll gradient and the tube was centrifuged in a swing rotor at 800*g* for 30 min. Cells from the density boundary (Fig. 9) were collected by gentle aspiration and washed twice in AS. The cell composition of fractions was determined by phase-contrast microscopy. Part of the cells of each fraction was used for SDS electrophoresis, the other part was fixed with Bouin's fixing solution. To fix the *S. rustica* cells, they were placed on the slides (Metzel Gläser, SuperFrost^®^ Plus) for spreading during 30 min, then they were fixed with Bouin's fixing solution for 30 min and washed in AS, dH_2_O and 30%, 50%, and stored in 70% ethanol. To fix the *H.aurantium* cells, the cell suspension was placed in Bouin's fluid for 30 min, then sequentially washed in AS, dH_2_O and 30%, 50%, and stored in 70% methanol at + 4 °C. To assess the purity of cell separation, fixed cells from each fraction were washed in dH_2_O and then applied to glass slides. Each fraction was stained with hematoxylin and eosin. The obtained preparations were analysed using a Leica DM6000 light microscope.

### Cells’ indirect immunolabeling with AB to p48

The cells of *S. rustica* spreaded on the slides were rehydrated for 10 min in dH_2_O and 10 min in TBST (TBST: 25 mM Tris–HCl pH 7.5; 130 mM NaCl; 0.05% Tween20). Fixed *H. aurantium* cell fractions in 70% methanol were centrifuged at 900*g* for 3 min. The cell pellet was resuspended in dH_2_O, followed by centrifugation at 900*g* for 3 min twice. Then, cell suspensions of each fraction in dH_2_O were placed on glass slides (X-tra Adhesive, Leica) and left to air dry overnight. The cells were then rehydrated for 5 min in dH_2_O and 10 min in TBST. After that, cells were permeabilised for 30 min RT using 0.4% (v/v) Triton X-100 (Fisher Scientific, Waltham, MA, USA) in TBST, washed three times in TBST and incubated for 1 h RT in a blocking buffer (2% BSA in TBST). Samples were then incubated for 1 h with primary polyclonal AB to p48—GPαP48Sr—diluted 1:2000 in TBST. As a negative control, primary antibody was replaced by TBST. After washing in TBST the cells were incubated for 1 h RT with secondary AB conjugated with horseradish peroxidase (RαGP-HRP, Sigma-Aldrich, #A5545) at a dilution of 1:500 in TBST. For visualisation of labelled material 0.35 mg/ml 3,3′diaminobenzidine (DAB, Sigma-Aldrich, # D5637) and 0.03% hydrogen peroxide were used. Cell fractions of *S. rustica* were also stained with hematoxylin and then embedded in dammar resin. Imaging was performed with a Leica DM6000 light microscope (Germany).

### SDS-PAGE and immunoblot

The cell pellet after fractionation in Percoll density gradient was mixed with 1 × Standard Laemmli buffer (25 mM Tris–HCl pH 6.8; 10% glycerol; 2% SDS; 5% β-mercaptoetanol) and boiled for 5 min. SDS-PAGE was performed in 12% polyacrylamide gels with Unstained or Prestained Protein MW marker (Thermo Scientific Broad Range Unstained #26630, BioRad Prestained All Blue #1610393, Spectra™ Multicolor #26634) and stained with Coomassie BB G-250 (Biolot, Russia) or used to transfer proteins to a nitrocellulose membrane 0.2 μm or 0.45 µm (BioRad, #1620071, #1620117) for the Western blot^91^. Staining of *S. rustica* proteins was carried out as described previously^48,49^. For *H. aurantium* proteins the membrane was blocked with 3% solution of bovine serum albumin (BSA) in 1xPBS, 0.05% Tween for 1.5 h at RT and incubated with primary AB (GPαp48Sr 1:5000 in PBS-Tween) with the addition of 3% BSA overnight in + 10 °C. The membrane was washed three times for 10 min with PBS-Tween and incubated with the secondary AB conjugated with alkaline phosphatase (GαGP-AP, Sigma-Aldrich, #A5062) for 2 h at a dilution of 1: 10,000 in PBS-Tween. For visualisation of labelled proteins were used BCIP (Fermentas, #R0822) and NBT (Fermentas, #R0842) according to the manufacturer’s recommendations.

### Primary transcriptome assembly and cloning of tuphoxin cDNA

Total blood cells of ascidian *S. rustica* were collected as described in section “Blood Collection”. Cells were centrifuged at 900*g* for seven minutes, supernatant was discarded and cell pellet frozen in liquid nitrogen. Total RNA was extracted by ExtractRNA kit (Evrogen, Russia) according to manufactures instructions but modified by addition of betamercaptoethanol to 5% at the first step and treated with DNAse I (Thermo Fisher Scientific) according to manufactures instructions. The RNA quality control, polyA RNA extraction with NEBNext^®^ Poly(A) mRNA Magnetic Isolation Module(NEB E7490, New England Biolabs, UK), mRNA library preparation with NEBNext^®^ Ultra™ II Directional RNA Library Prep (NEB E7760, New England Biolabs, UK) and sequencing was carried out at the research resource centre "Biobank" of Saint-Petersburg State University (St.Petersburg, Russia). Sequencing was performed on an Illumina Hiseq 4000 platform to obtain paired-end reads. Raw reads quality control was verified with FastQC v 0.11.7 (http://www.bioinformatics.babraham.ac.uk/projects/fastqc/). Raw reads were submitted to Sequence Read Archive (BioProject ID:PRJNA772663). To obtain clean reads, we removed adaptors and unpaired reads with Trimmomatic v. 0.36 91. The transcriptome was assembled with rnaSPAdes v. 3.11.1 92 with default parameters with *S. canopus* transcripts used as reference contigs. A total of 37,144,302 transcriptomic paired-end reads were generated for *S. rustica*; 36,175,852 of them passed quality filters and trimming and yielded in 307,180 transcripts. The assessment of transcripts’ completeness was evaluated with BUSCO (https://busco.ezlab.org/) with Metazoan lineage dataset. The exhaustiveness of *S. rustica* transcriptome assembly compared to other *Styela* species^92^ is summarised in Table 3. Assembly comprising 199,431 transcripts longer than 200 bp is available at https://github.com/AnnaSolovyeva/Styela-rustica.

**Table 3:** BUSCO evaluation of *S. rustica* transcriptome compared to other *Styela sp.*

BUSCO parameter	*S. rustica*	*S. canopus*	*S. plicata*
Complete BUSCOs (C)	872	757	912
Complete and single-copy BUSCOs (S)	503	642	882
Fragmented BUSCOs (F)	53	115	30
Complete and duplicated BUSCOs (D)	369	104	15
Missing BUSCOs (M)	29	93	27
Total BUSCO groups searched (metazoan lineage)	954	954	954

Clean reeds of *S. rustica* transcriptome were mapped once again on assembled tuphoxin transcript in order to get longer sequence. This sequence was used to design primers for PCR amplification of tuphoxin transcript (p48_F:gtctctgtttcatacactcatgtataaaacctg, p48_R:gcactgcgaggttgtcata). Total RNA of blood cells was reverse-transcribed with MINT cDNA synthesis kit (Evrogen, Russia). PCR product was amplified on the matrix of blood cells cDNA pretreated with DNAse I (New England Biolabs (UK), #M0303L) and cloned in pTZ75 r/t vector (Thermo Scientific, #K1214). Sanger sequencing was carried out at the research resource centre "Biobank" of Saint-Petersburg State University (St.Petersburg, Russia).

### MALDI TOF/TOF mass spectrometry

A protein bands corresponding to a certain molecular weight was cut from the polyacrylamide gel and digested with trypsin (Trypsin Gold, Promega) Ttyptic digests were dissolved in 1% formic acid, filtered through of 0.22 μm filter, and subjected to chromatographic separation using a Milichrom-A02 system on a BioBasic-18 reversed-phase column (5 μm, 300 Å, 50 × 1 mm, Thermo Fisher). Elution was carried out with gradient of eluent B to A from 2 to 45% and flow rate of 50 μl/min, where A is 5% acetonitrile, 0.1% trifluoroacetic acid (TFA) and B is 60% acetonitrile, 0.1% TFA. The eluate was mixed with a matrix solution (CHSA, 10 mg/ml) and automatically applied to a MALDI target (260 spots) using a microfraction collector. The fractionated samples were analysed with a TOF/TOF 5800 System (SCIEX) instrument operated in the positive ion mode. The MALDI stage was set to continuous motion mode. MS data was acquired at 2600 laser intensity with 800 laser shots/spectrum (200 laser shots/sub-spectrum) and MS/MS data were acquired at 3600 laser intensity with a DynamicExit algorithm and a high spectral quality threshold or a maximum of 1000 laser shots/spectrum (250 laser shots/sub-spectrum). Up to 25 top precursors with S/N > 40 in the mass range 750–4000 Da were selected from each spot for MS/MS analysis. The Protein Pilot 5.0.1 software (SCIEX, Darmstadt, Germany) with the Paragon algorithm in thorough mode was used for the MS/MS spectra search against the pooled protein database comprising 89,591 protein-coding sequences predicted by Transdecoder v.5.5.0^93^ from assembly datasets of ascidians *S. rustica* (this paper), *S. canopus*^92^ and *H. aurantium*^50^. Carbamidomethyl cysteine was set as a fixed modification. The database also incorporated a list of common contaminants.

### Analysis of sequences similar to *Tuphoxin*

Amino acid sequences of proteins similar to *Tuphoxin* belonging to *Styela* and *Halocynthia* species were aligned using ClustalX^94^. Signal peptides were predicted in SignalP-5.0^95^ and trimmed for subsequent analysis. Molecular weight and isoelectric point of the predicted mature protein sequences were calculated using “Compute pI/Mw” tool from the ExPASy resource of the Biological Server at the Swiss Institute for Bioinformatics (http://www.expasy.org). The presence of conserved functional domains in aa sequences was predicted using the Conserved Domain search tool of the NCBI^96^. For *C. intestinalis* sequence XM_026835721.1, transcript variant X1, exons boundaries were retrieved from the NCBI database. In order to determine the active site residues, sequence of tyrosinase domain was searched in the UniRef database (version 30_2020_06) by HHblits 3.2.0 at the Bioinformatics Toolkit resource (https://toolkit.tuebingen.mpg.de/). The aligned regions of the sequences found were redirected to HHpred 3.2.0 for searches in the database of structures — Protein Data Bank (version PDB_mmCIF70_17_May). Localisation of the active site aas were determined by alignment with the most similar protein — *Asperugillus oryzae* tyrosinase (6JU5_A).

### Phylogeny construction

Homologues for phylogeny construction were searched in several databases. Isolated sequence of tyrosinase domain encoded by *S. canopus* transcript was searched against nucleotide databases : nr (Release 240, October 15, 2020), EST and TSA (searching date November 2020) using tBLASTn and BLASTp algorithm^97^. In order to search sequences in specialised ascidians databases we used Aniseed web portal (https://www.aniseed.cnrs.fr/)^98^, transcriptomic databases of ascidians *S. canopus*, *S. plicata, S. clava* provided by Alie and coauthors^92^ and draft transcriptome of *S. rustica* blood cells sequenced de novo (section Transcriptome). Artropod-like POs of ascidians were derived from GenBank according to ID from the literature (Supplementary Table S2). For phylogeny construction aa sequences were aligned with MAFFT^99^. For arthropod-like POs alignment was then manually curated. Then sequences were filtered to 90% identity in HHfilter^100^, informative regions were selected by GBLOCKs 0.91b with the lest stringent conditions^101^. Substitution model was chosen using MEGA-X software^102^. Maximum Likelihood (ML) tree was constructed in IQtree web server^103^ with LG + G^104^ model and empirical state frequencies computed from alignment, we used ultrafast bootstrap branch support after 1000 replicates. Parallel phylogenetic analysis with the same data was carried out with BEAST software (v1.10.4)^105^. Three independent runs of MCMC chains 10 million iterations each, burn-in first 2.5 million and sampling every 1000 iteration. Maximum clade credibilty tree was constructed using TreeAnnotator (v1.10.4). All consensus trees were visualised in FigTree (v1.4.4) (http://tree.bio.ed.ac.uk/).

## Supplementary Information

Supplementary Information.

## Data Availability

The dataset supporting the conclusions of this article are available in the several repositories. Raw data for *S. rustica* are available at NCBI sequencing read archive (BioProject ID : P RJNA772663)^106^ and assembly is available at GitHub repository https://github.com/AnnaSolovyeva/Styela-rustica^107^. Data for *S. canopus, S. clava* and *S. plicata* were downloaded from GitHub repository https://github.com/AlexAlie/styelida^92^. Data for *H. aurantium* was downloaded from Aniseed web portal https://www.aniseed.cnrs.fr/^98^. All other data are available from NCBI genbank (https://www.ncbi.nlm.nih.gov/genbank/).

## Abbreviations

AAS: Amino acid substitutions
AB: Antibodies
BI: Bayesian inference
BSA: Bovine serum albumin
CuOx: Domain of cupredoxin family
ECM: Extracellular matrix
Hc: Haemocyanin
MALDI: Matrix assisted laser desorption ionization
ML: Maximum likelihood
LPS: Lipopolysaccharides
PO: Phenol oxidase
TFA: Trifluoroacetic acid
TSP1: Thrombospondin first type repeat
Tyr: Tyrosinase
