# Supplementary material for: New putative phenol oxidase in ascidian blood cells
Source: Sci Rep. 2022 Aug 22;12:14326. doi: 10.1038/s41598-022-18283-9 (PMC9395347; doi:10.1038/s41598-022-18283-9)
Supplement: Supplementary file 1 — Supplementary Information. [file 41598_2022_18283_MOESM1_ESM.pdf]

New putative phenoloxidase in ascidian blood cells

Daugavet M.A.[1*], Dobrynina M.I.[1], Shaposhnikova T.G.[2], Solovyeva A.I.[1,3], Mittenberg A.[1], Shabelnikov S.V.[1], Babkina I.[2], Grinchenko A.[4], Ilyaskina D.[4,5], Podgornaya O.I.[1,2]

1 Institute of Cytology of Russian Academy of Sciences, St. Petersburg, Russia.
2 Saint-Petersburg State University, St. Petersburg, Russia.
3 Zoological Institute of Russian Academy of Sciences, St. Petersburg, Russia.
4 A.V.Zhirmunsky National Scientific Center of Marine Biology, Vladivostok, Russia.
5 Vrije Universiteit Amsterdam, HV The Netherlands.

*Corresponding author: E-mail: ka6tanka@yandex.ru

Supplementary information

```
Domains
Sru_Tuph MKKYKKDTSNFGFHNAAGTHVFPYQCMGPDGNMAGCCYHGARLNFAMWHRAELLNFEEGL
peptides --------------------------------------------------AELLNFEEGL

Domains                              TYR
Sru_Tuph TRHLKDKTLGLPYWDWLKYPEPPALVWSKKLYGKENPFTSMTIRYDTTPHTLTQRSPTVY
peptides TR--------------------------------ENPFTSMTIRYDTTPHTLTQRSPTVY

Domains
Sru_Tuph PFMVKVSKDNYYVAQKLMQSHTIHEFSEALENSHNTIHAGICWGKESAAGKCTYSMGSLT
peptides PFMVK-------------------------------------------------------

Domains
Sru_Tuph YAGFDPVFFFHHANVDRLFSVFQNWMSAHGEEAWTRQSVLEPQENIFHF
peptides -------------------------------------------------
```

Supplementary Figure S1. Peptides of 26 kDa band of *Styela rustica*.

Alignment of tryptic peptides from 26 kDa protein band with *in silico* translated *Tuphoxin* transcript of *S. rustica* (Sru_*Tuph*). Tryptic peptides are merged in longer sequences. Predicted conserved domain of tyrosinase (TYR) is marked by orange rectangle.

```
Domains
Sca_Tuph KDSSTFGFHNAGGTHEFPFQCMGSSGEDKGCCYHGARLNFVMWHRAELLNFEEGLSRHLR
peptides ---------------------------------------------AELLNFEEGLTR

Domains                               TYR
Sca_Tuph DKTLGLPYWDWLTYPNPPPLVWSKKLYNKANPFTSGLVRYDTKPHTLTQRSPTVDPFMVK
peptides --------------------------------------------------SPTVDPFMVK

Domains
Sca_Tuph MSKDNYYVARKLMQSHSIHEFSEALENSHNTIHSSICWGAQQKAGKCEFSMGGLTYAAFD
peptides ------------------------------------------------------------

Domains
Sca_Tuph PVFFFHHTNVDRLFSVFQNWMTNNNEVAWTRQSVLEPQENIFHFNQPFLPFNNRSLTPFK
Peptides ------------------------------------------------------------
```

Supplementary Figure S2. Peptides of 26 kDa band of *Styela canopus*.

Alignment of tryptic peptides from 26 kDa band with *in silico* translated *Tuphoxin* transcript of *Styela canopus* (Sca_*Tuph*). Tryptic peptides are merged in longer sequences, with aa substitutions compared to translated transcript highlighted in yellow. Predicted conserved domain of tyrosinase (TYR) is marked by orange rectangle.

```
Hau_Tuph1 QRDTRNKLVERLEGPEDIESEESPFRDDRENSLDEDEDNVLVYKGEHDHLTKALPGATCE  60
Peptides  ------------------------------------------------------------

Hau_Tuph1 AGYQIKPGCIFCQDIDECTTGTEKTLTVDQLLNADSDGLNNEVTKDQILTFHSEKALAIG  120
Peptides  ------------------------------------------------------------

Hau_Tuph1 KPLTPVKLYYRQPCSTLGSTMVQNLHLNKDKIPDMIITYVCVRSGSTNMYRIKSVFDKEA  180
Peptides  ------------------------------------------------------------

Domains                       TSP
Hau_Tuph1 KWTEWTTWTACKPTIWGGLQTRARICKKGLVGYGKCLGSASAYRECYRGKKEALRVRREA  240
Peptides  ------------------------------------------------------------

Domains                                         *                   *
Hau_Tuph1 NTLTTAQKSDLIQAMKKYKDDLSTYGYHQAAGTHFWPNECVKPNGVSGACCYHGAKVNFV  300
Peptides  ------------------------------------------------------------

Domains     *                            TYR
Hau_Tuph1 MWHRAELLYYEQGLTRHLKDKKLGAPYWDWLVYPNPPQLVWAKTIGGKPNPFAGSKIRYD  360
Peptides  ------------------------------------------KTIGGKPNPFAGSKI---

Domains                                                        *   *
Hau_Tuph1 LPVVISTARSPAVSSSSVSMAKSIFQTITSKLARSHMIHEYSESLESSHNNIHSAVCHAD  420
Peptides  ------------------------------------------------------------

Domains                           *
Hau_Tuph1 TGCDYSMGELEYAAYDPIFFFHHANVDRLFSVFQSHMVKKKDMAWTRQGFLEPSENIYHF  480
Peptides  ------------------------------------------------------------

Hau_Tuph1 NEPYQPFRNKTLTPFKKLRVANSIADLLYYEQLLGYKYDSLKAHNTKLNTKDAIEEKSHV  540
Peptides  -----------------RVANSIADLLYYEQLLGYKYDSLKA------------------

Hau_Tuph1 PTKTDVVVLAQYEQSTGLNVKMRYFYAGGTTPLPPCASIADSNAAYLGTLTILGSRKERA  600
Peptides  ------------------------------------------------------------

Hau_Tuph1 WTAKQPTLLDITSKLTELEATLTSNIKIKACYTHGMGCQSKCKEIFATVRTKIPAPQTIL  660
Peptides  ------------------------------------------------------------

Domains
Hau_Tuph1 YPSNIDVDIIKFRWDVGHRMPIGKIVVNAGKEYIYFWGKEAKNVVQVGRTCYDSCSAMGG  720
Peptides  ------------------------------------------------------------

Domains             CuOx
Hau_Tuph1 KTVNCPYGCALPAGSHFFVNKKYCKSGLKLGILSVI  756
Peptides  ------------------------------------
```

Supplementary Figure S3. Peptides of 48 kDa band of *Halocynthia aurantium.*

Alignment of tryptic peptides from 48 kDa band of morula cells with *in silico* translated *Tuphoxin* transcript of *H. aurantium* (Hau_*Tuph1*). Tryptic peptides are merged in longer sequences. Predicted conserved domains are marked by rectangles: green (thrombospondin first type repeat - TSP1), orange (tyrosinase - TYR) and blue (domain of cupredoxin family – CuOx). Asterisks (*) indicate the active site amino acids.

```
Hau_Tuph1 QRDTRNKLVERLEGPEDIESEESPFRDDRENSLDEDEDNVLVYKGEHDHLTKALPGATCE  60
Peptides  ------------------------------------------------------------

Hau_Tuph1 AGYQIKPGCIFCQDIDECTTGTEKTLTVDQLLNADSDGLNNEVTKDQILTFHSEKALAIG  120
Peptides  -------------------------------------------------------ALAIG

Hau_Tuph1 KPLTPVKLYYRQPCSTLGSTMVQNLHLNKDKIPDMIITYVCVRSGSTNMYRIKSVFDKEA  180
Peptides  KPLTPVK----QPCSTLGSTMVQNLHLNKDKIPDMIITYVCVR--------IKSVFDKEA

Domains                       TSP 1
Hau_Tuph1 KWTEWTTWTACKPTIWGGLQTRARICKKGLVGYGKCLGSASAYRECYRGKKEALRVRREA  240
Peptides  K--------------------------KGLVGYGKCLGSASAYR-------------REA

Domains
Hau_Tuph1 NTLTTAQKSDLIQAMKKYKDDLSTYGYHQAAGTHFWPNECVKPNGVSGACCYHGAKVNFV  300
Peptides  NTLTTAQKSDLIQAMKKYKDDLSTYGYHQAAG------------GVSGACCYHGAKVNFV

Domains                         TYR
Hau_Tuph1 MWHRAELLYYEQGLTRHLKDKKLGAPYWDWLVYPNPPQLVWAKTIGGKPNPFAGSKIRYD  360
Peptides  MWHRAELLYYEQGLTR---------------------------TIGGKPNPFAGSKIRYD

Domains
Hau_Tuph1 LPVVISTARSPAVSSSSVSMAKSIFQTITSKLARSHMIHEYSESLESSHNNIHSAVCHAD  420
Peptides  LPVVISTARSPAVSSSSVSMAKSIFQTITSK-----------------------------

Domains
Hau_Tuph1 TGCDYSMGELEYAAYDPIFFFHHANVDRLFSVFQSHMVKKKDMAWTRQGFLEPSENIYHF  480
Peptides  ----------------------------LFSVFQSHMVK--------QGFLEPSENIYHF

Hau_Tuph1 NEPYQPFRNKTLTPFKKLRVANSIADLLYYEQLLGYKYDSLKAHNTKLNTKDAIEEKSHV  540
Peptides  NEPYQPFR----------------------------------------------------

Hau_Tuph1 PTKTDVVVLAQYEQSTGLNVKMRYFYAGGTTPLPPCASIADSNAAYLGTLTILGSRKERA  600
Peptides  ------------------------------------------------------------

Hau_Tuph1 WTAKQPTLLDITSKLTELEATLTSNIKIKACYTHGMGCQSKCKEIFATVRTKIPAPQTIL  660
Peptides  ------------------------------------------------------------

Domains
Hau_Tuph1 YPSNIDVDIIKFRWDVGHRMPIGKIVVNAGKEYIYFWGKEAKNVVQVGRTCYDSCSAMGG  720
Peptides  ------------------------------------------------------------

Domains       CuOx
Hau_Tuph1 KTVNCPYGCALPAGSHFFVNKKYCKSGLKLGILSVI  756
Peptides  ------------------------------------
```

Supplementary Figure S4. Peptides of 35 kDa band of *Halocynthia aurantium.*

Alignment of tryptic peptides from 35 kDa band of morula cells with *in silico* translated *Tuphoxin* transcript of *H. aurantium* (Hau_*Tuph1*). Tryptic peptides are merged in longer sequences. Predicted conserved domains are marked by rectangles: green (thrombospondin first type repeat - TSP1), orange (tyrosinase - TYR) and blue (domain of cupredoxin family – CuOx).

```
Hau_Tuph1 QRDTRNKLVERLEGPEDIESEESPFRDDRENSLDEDEDNVLVYKGEHDHLTKALPGATCE  60
Hau_Tuph2 ------------------------------------------------------------  0
Peptides  ------------------------------------------------------------

Hau_Tuph1 AGYQIKPGCIFCQDIDECTTGTEKTLTVDQLLNADSDGLNNEVTKDQILTFHSEKALAIG  120
Hau_Tuph2 ------------------------------------------------------------  0
Peptides  -------------------------------------------------------ALAIG

Hau_Tuph1 KPLTPVKLYYRQPCSTLGSTMVQNLHLNKDKIPDMIITYVCVRSGSTNMYRIKSVFDKEA  180
Hau_Tuph2 --------------------MVQNLHLNKDKIPDMIITYVCVRSGSTNMYRIKSVFDKE-  39
Peptides  KPLTPVK-----------------------------------------------------

Domains                       TSP 1
Hau_Tuph1 KWTEWTTWTACKPTIWGGLQTRARICKKGLVGYGKCLGSASAYRECYRGKKEALRVRREA  240
Hau_Tuph2 ----------------------------------------------------ALRVRREA
Peptides  ------------------------------------------------------------

Domains                                         *                  *
Hau_Tuph1 NTLTTAQKSDLIQAMKKYKDDLSTYGYHQAAGTHFWPNECVKPNGVSGACCYHGAKVNFV  300
Hau_Tuph2 NTLTTAQKSDLIQAMKKYKDDLSTYGYHQAAGTHFWPNECVKPNGVSGACCYHGAKVNFV  107
Peptides  ------------------------------------------------------------

Domains     *
Hau_Tuph1 MWHRAELLYYEQGLTRHLKDKKLGAPYWDWLVYPNPPQLVWAKTIGGKPNPFAGSKIRYD  360
Hau_Tuph2 MWHRAELLYYEQGLTRHLKDKKLGAPYWDWLVYPNPPQLVWAKTIGGKPNPFAGSKIRYD  167
Peptides  ----AELLYYEQGLTR--------------------------------------------

Domains                          TYR                         *   *
Hau_Tuph1 LPVVISTARSPAVSSSSVSMAKSIFQTITSKLARSHMIHEYSESLESSHNNIHSAVCHAD  420
Hau_Tuph2 LPVVISTARSPAVSSSSVSMAKSIFQTITSKLARSHMIHEYSESLESSHNNIHSAVCHAD  227
Peptides  ------------------------------------------------------------

Domains                       *
Hau_Tuph1 TGCDYSMGELEYAAYDPIFFFHHANVDRLFSVFQSHMVKKKDMAWTRQGFLEPSENIYHF  480
Hau_Tuph2 TGCDYSMGELEYAAYDPIFFFHHANVDRLFSVFQSHMVKKKDMAWTRQGFLEPSENIYHF  287
Peptides  ------------------------------------------------------------

Hau_Tuph1 NEPYQPFRNKTLTPFKKLRVANSIADLLYYEQLLGYKYDSLKAHNTKLNTKDAIEEKSHV  540
Hau_Tuph2 NEPYQPFRNKTLTPFKKLRVANSIADLLYYEQLLGYKYDSLKAHNTKLNTKDAIEEKSHV  347
Peptides  ---------------------------------------------------------SHV

Hau_Tuph1  PTKTDVVVLAQYEQSTGLNVKMRYFYAGGTTPLPPCASIADSNAAYLGTLTILGSRKERA  600
Hau_Tuph2  PTKTDVVVLAQYEQSTGLNVKMRYFYAGGTTPLPPCASIADSNAAYLGTLTILGSRKERA  407
Peptides   PTKTDVVVLAQYEQSTGLNV----------------------------------------

Hau_Tuph1 WTAKQPTLLDITSKLTELEATLTSNIKIKACYTHGMGCQSKCKEIFATVRTKIPAPQTIL  660
Hau_Tuph2 WTAKQPTLLDITSKLTELEATLTSNIKIKACYTHGMGCQSKCKEIFATVRTKIPAPQTIL  467
Peptides  ---KQPTLLDITSKLTELEATLTSNIK----------------------TKIPAPQTIL

Domains
Hau_Tuph1  YPSNIDVDIIKFRWDVGHRMPIGKIVVNAGKEYIYFWGKEAKNVVQVGRTCYDSCSAMGG  720
Hau_Tuph2  YPSNIDVDIIKFRWDVGHRMPIGKIVVNAGKEYIYFWGKEAKNVVQVGRTCYDSCSAMGG  527
Peptides   YPSNIDVDIIK--WDVGHR-----------EYIYFWGK---NVVQVGR-----------

Domains       CuOx
Hau_Tuph1  KTVNCPYGCALPAGSHFFVNKKYCKSGLKLGILSVI  756
Hau_Tuph2  KTVNCPYGCALPAGSHFFVNKKYCKSGLKLGILSVI  563
Peptides   -TVNCPYGCALPAGSHFFVNK---------------
```

Supplementary Figure S5. Alignment of two *Tuphoxin* transcripts of *Halocynthia aurantium.*

Alignment of tryptic peptides from 26 kDa band of morula cells with two *in silico* translated *Tuphoxin* transcripts of *H. aurantium* (Hau_*Tuph1* and Hau_*Tuph2*). Tryptic peptides are merged in longer sequences. Predicted conserved domains are marked by rectangles: green (thrombospondin first type repeat - TSP1), orange (tyrosinase - TYR) and blue (domain of cupredoxin family – CuOx). Asterisks (*) indicate the active site amino acids.

```
Hau_Tuph1 WTAKQPTLLDITSKLTELEATLTSNIKIKACYTHGMGCQSKCKEIFATVRTKIPAPQTIL  660
Peptides  --------------LTELEATLTSNIK---------------------------------

Domains
Hau_Tuph1 YPSNIDVDIIKFRWDVGHRMPIGKIVVNAGKEYIYFWGKEAKNVVQVGRTCYDSCSAMGG  720
Peptides  -------------------------------EYIYFWGK---------------------

Domains           CuOx
Hau_Tuph1 KTVNCPYGCALPAGSHFFVNKKYCKSGLKLGILSVI  756
Peptides  ------------------------------------
```

Supplementary Figure S6. Peptides of 26 kDa band of *Halocynthia aurantium* hyalinocytes.

Alignment of tryptic peptides from 26 kDa band of hyalinocytes with *in silico* translated *Tuphoxin* transcript of *H. aurantium* (Hau_*Tuph1*). Predicted conserved domain of cupredoxin family (CuOx) is marked by blue rectangle.

```
Sru_Tuph  ------------------------------------------------------------  0
Sca_Tuph  MREALVITLLWVCVAVTQVEGRKKTCAVNFGGCDKSRGLCWNTDGGKSSMCSCKAGYRFR  60
Hau_Tuph1 ------------------------------------------------------------  0
Hau_Tuph2 ------------------------------------------------------------  0

Domains             +  +      EGF_CA1          +
Sru_Tuph  ------------------------------------------------------------  0
Sca_Tuph  LPHTRRGRCVDINECRANLHSCDTKNGGCTNTIGSHRCYCKKGFVLDYTKTKCLRRKHIK  120
Hau_Tuph1 ------------------------------------------------------------  0
Hau_Tuph2 ------------------------------------------------------------  0

Domains                                                             +  +
Sru_Tuph  ------------------------------------------------------------  0
Sca_Tuph  GTCRGRRCGRCRNRFRYDAEDEDASKFLLRLLRRRKGRRCVCEPGFAKRRGCKYCQDINE  180
Hau_Tuph1 ------------------------------------------------------------  0
Hau_Tuph2 ------------------------------------------------------------  0

Domains    EGF_CA2               +
Sru_Tuph  ----------------------------------CGFKCYEPTKRPSTCNSESFQSSSLS  26
Sca_Tuph  CRLRISMCDLRRGYCHNSDGGYFCSCRKGYVLGPCGFKCYRPKKRPSDCNSEIFKSSSLA  240
Hau_Tuph1 ------------------------------------------------------------  0
Hau_Tuph2 ------------------------------------------------------------  0

Sru_Tuph  KGDSDGLNDETTKAVLLDYITEKIAKSGGKLSLFHTLMYKTCKTGGAKRVQRFDVDGDGK  86
Sca_Tuph  KGDSDGLNDETTKAVLLDYITEKIAKAGGRLTLFHTLMYKTCKTGQKKRIQRFDINRDGK  300
Hau_Tuph1 ------------------------------------------------MVQNLHLNKDKI  12
Hau_Tuph2 ------------------------------------------------MVQNLHLNKDKI  12
                                                           :*.:.:: *

Domains                                              TSP 1
Sru_Tuph  TDIIINYVCVGL----KWTVKNVFDTEAKWETWSCWNTCKPTCSGGIQTRTRICKHGLVG  142
Sca_Tuph  DDLVIHYVCVGN----KWTVKAVYEEEARWAPWTCWTSCKPTCSGGIQTRVRICKNGLVG  356
Hau_Tuph1 PDMIITYVCVRSGSTNMYRIKSVFDKEAKWTEWTTWTACKPTIWGGLQTRARICKKGLVG  72
Hau_Tuph2 PDMIITYVCVRSGSTNMYRIKSVFDKE---------------------------------  39
           *::* ****       : :* *:: *

Domains
Sru_Tuph  EGRCKGPATEYKECYTGRKCKLRVRREAHSLNRVQKKDLIQAMKKYKKDTSNFGFHNAAG  202
Sca_Tuph  EGKCKGRATQYKTCYTGKKCPLRVRREAHSLNTRQKKDLIQAMEKYKKDSSTFGFHNAGG  416
Hau_Tuph1 YGKCLGSASAYRECYRGKKEALRVRREANTLTTAQKSDLIQAMKKYKDDLSTYGYHQAAG  132
Hau_Tuph2 --------------------ALRVRREANTLTTAQKSDLIQAMKKYKDDLSTYGYHQAAG  79
                               *******::*.  **.******:***.* *.:*:*:*.*

Domains
Sru_Tuph  THVFPYQCMGPDGNMAGCCYHGARLNFAMWHRAELLNFEEGLTRHLKDKTLGLPYWDWLK  262
Sca_Tuph  THEFPFQCMGSSGEDKGCCYHGARLNFVMWHRAELLNFEEGLSRHLRDKTLGLPYWDWLT  476
Hau_Tuph1 THFWPNECVKPNGVSGACCYHGAKVNFVMWHRAELLYYEQGLTRHLKDKKLGAPYWDWLV  192
Hau_Tuph2 THFWPNECVKPNGVSGACCYHGAKVNFVMWHRAELLYYEQGLTRHLKDKKLGAPYWDWLV  139
          ** :* :*:  .*   .******::**.******** :*:**:***:**.** ******

Domains                              TYR
Sru_Tuph  YPEPPALVWSKKLYGKENPFTSMTIRYDTTPHTLTQRSPTVYPFMVKVSKDNYY-VAQKL  321
Sca_Tuph  YPNPPPLVWSKKLYNKANPFTSGLVRYDTKPHTLTQRSPTVDPFMVKMSKDNYY-VARKL  535
Hau_Tuph1 YPNPPQLVWAKTIGGKPNPFAGSKIRYDLPVVISTARSPAVSSSSVSMAKSIFQTITSKL  252
Hau_Tuph2 YPNPPQLVWAKTIGGKPNPFAGSKIRYDLPVVISTARSPAVSSSSVSMAKSIFQTITSKL  199
          **:** ***:*.: .* ***:.  :***      * ***:*    *.::*. :  :: **

Domains
Sru_Tuph  MQSHTIHEFSEALENSHNTIHAGICWGKESAAGKCTYSMGSLTYAGFDPVFFFHHANVDR  381
Sca_Tuph  MQSHSIHEFSEALENSHNTIHSSICWGAQQKAGKCEFSMGGLTYAAFDPVFFFHHTNVDR  595
Hau_Tuph1 ARSHMIHEYSESLESSHNNIHSAVCHA----DTGCDYSMGELEYAAYDPIFFFHHANVDR  308
Hau_Tuph2 ARSHMIHEYSESLESSHNNIHSAVCHA----DTGCDYSMGELEYAAYDPIFFFHHANVDR  255
           :** ***:**:**.***.**:.:* .       * :*** * **.:**:*****:****

Domains
Sru_Tuph  LFSVFQNWMSAHGEEAWTRQSVLEPQENIFHFNEPYLPFRNKTLTPFKKLQELASIQNMF  441
Sca_Tuph  LFSVFQNWMTNNNEVAWTRQSVLEPQENIFHFNQPFLPFNNRSLTPFKKLHALSTIQDLF  655
Hau_Tuph1 LFSVFQSHMVKKKDMAWTRQGFLEPSENIYHFNEPYQPFRNKTLTPFKKLRVANSIADLL  368
Hau_Tuph2 LFSVFQSHMVKKKDMAWTRQGFLEPSENIYHFNEPYQPFRNKTLTPFKKLRVANSIADLL  315
          ******. *  : : *****..***.***:***:*: **.*::*******:   :* :::

Sru_Tuph  YYRELLGYRYDNLAV---------------------------------------------  456
Sca_Tuph  FNRELLGYRYDSLKVPKEKKTKKVGDEEKAHIPSSGDKLMLGQFERNIRTNVKITYFFAN  715
Hau_Tuph1 YYEQLLGYKYDSLKAHNTKLNTKDAIEEKSHVPTKTDVVVLAQYEQSTGLNVKMRYFYAG  428
Hau_Tuph2 YYEQLLGYKYDSLKAHNTKLNTKDAIEEKSHVPTKTDVVVLAQYEQSTGLNVKMRYFYAG  375
          : .:****:**.* .

Sru_Tuph  ------------------------------------------------------------  456
Sca_Tuph  KIKGFKDIKTCSKLNEDETRYLASAQILGSKRERAWKSRQPTVVDISNLFGKLTWQQRRT  775
Hau_Tuph1 G---TTPLPPCASIADSNAAYLGTLTILGSRKERAWTAKQPTLLDITSKLTELE------  479
Hau_Tuph2 G---TTPLPPCASIADSNAAYLGTLTILGSRKERAWTAKQPTLLDITSKLTELE------  426

Sru_Tuph  ------------------------------------------------------------  456
Sca_Tuph  SGLSATSNIQIRACYTHNLSCRKNCKQIRSRVKKLIPSPQLILYPKNIRTDIQKIEWAND  835
Hau_Tuph1 --ATLTSNIKIKACYTHGMGCQSKCKEIFATVRTKIPAPQTILYPSNIDVDIIKFRWDVG  537
Hau_Tuph2 --ATLTSNIKIKACYTHGMGCQSKCKEIFATVRTKIPAPQTILYPSNIDVDIIKFRWDVG  484

Domains                                       CuOx
Sru_Tuph  ------------------------------------------------------------  456
Sca_Tuph  VRLPTWTIDAVSGTRWFVFWGSEAKNVVQVASRRAYLTCNTRGARKVRCETACVLPSGIH  895
Hau_Tuph1 HRMPIGKIVVNAGKEYIYFWGKEAKNVVQVG-RTCYDSCSAMGGKTVNCPYGCALPAGSH  596
Hau_Tuph2 HRMPIGKIVVNAGKEYIYFWGKEAKNVVQVG-RTCYDSCSAMGGKTVNCPYGCALPAGSH  543

Domains
Sru_Tuph  --------------------  456
Sca_Tuph  YFVHKTRCRRGMKLSVLAHL  915
Hau_Tuph1 FFVNKKYCKSGLKLGILSVI  616
Hau_Tuph2 FFVNKKYCKSGLKLGILSVI  563
```

Supplementary Figure S7. Alignment of *Tuphoxin* sequences belonging to *Styela rustica*, *S. canopus* and *Halocynthia aurantium*.

Alignment of four translated sequences of *Tuphoxins* belonging to *S. rustica* (Sru_*Tuph*), *S. canopus* (Sca_*Tuph*), and *H. aurantium* (Hau_*Tuph1*, Hau_*Tuph2*). Predicted conserved domains are marked by rectangles: grey (calcium-binding EGF-like – EGF_CA), green (thrombospondin first type repeat - TSP1), orange (tyrosinase - TYR), blue - cupredoxin family domain (CuOx).

A Adhesion G protein-coupled receptor B3 of *Homo sapiens* - O60242 (AGRB3_HUMAN)
Expect = 6e-10 Identities = 21/46 (46%)

```
TSP1    1   WETWSCWNTCKPTCSGGIQTRTRICKHGLVGEGRCKGPATEYKECY 46
            W+ WS W  C  TCS G Q R+R C     G   C+GP  E +ECY
AGRB3   403 WQEWSSWSQCSVTCSNGTQQRSRQCTAAAHGGSECRGPWAESRECY 448
```

B Zinc metalloproteinase nas-36 OS of *Caenorhabditis briggsae* - Q61EX6 (NAS36_CAEBR)
Expect = 3e-06 Identities = 23/53 (43%)

```
TSP1    1   WAPWTCWTSCKPTCSG-GIQTRVRICKNGLVGEGKCKGRATQYKTCYTGKKCP 52
            WA W  W+ C  TC G GI++RVR C+       KC+GR  ++ TC   K CP
NAS36   509 WADWGEWSMCSRTCGGCGIRSRVRSCRS-----KKCEGRRQEFGTCNL-KACP 555
```

Supplementary Figure S8. Results of similarity search for TSP1 domain.

Alignment of Tuphoxins sequences of TSP1 belonging to *S. rustica* (Sru_*Tuph*) (A) and *S. canopus* (Sca_*Tuph*) (B) with best hit sequences in UniProtKB/Swiss-Prot database.

Supplementary Table S1. Sequences of *Tuphoxin* homologues used for phylogeny construction.

Colour codes				
Ascidiacea	Annelida	Mollusca	Fungi	Cyanobacteria

Database ID		DB	Name	Species
Contig_28777	a)	de novo	**Sru_Tuphoxin**	*Styela rustica*
Contig_67444	a)	de novo		*Styela rustica*
Contig_7091		de novo	S.rustica.1	*Styela rustica*
Contig_7704		de novo	S.rustica.2	*Styela rustica*
Contig_15578		de novo	S.rustica.3	*Styela rustica*
SC_m.117002.p1		GitHub	**Sca_Tuphoxin**	*Styela canopus*
SC_m.152480.p1		GitHub	S.canopus.1	*Styela canopus*
SC_m.16402.p1		GitHub	S.canopus.2	*Styela canopus*
SC_m.86302.p1		GitHub	S.canopus.3	*Styela canopus*
SC_m.42250.p1		GitHub	S.canopus.4	*Styela canopus*
SC_m.26137.p1		GitHub	S.canopus.5	*Styela canopus*
AA49_m.7122.p1		GitHub	S.clava.1	*Styela clava*
AA49_m.7119.p1		GitHub	S.clava.2	*Styela clava*
AA49_m.30831.p1		GitHub	S.clava.3	*Styela clava*
AA49_m.87151.p1		GitHub	S.clava.4	*Styela clava*
AA49_m.2942.p1		GitHub	S.clava.5	*Styela clava*
AA49_m.30764.p1		GitHub	S.clava.6	*Styela clava*
AA49_m.72475.p1		GitHub	S.clava.7	*Styela clava*
AA49_m.3326.p1		GitHub	S.clava.8	*Styela clava*
Haaura.CG.MTP2014.S1292.g08050.02.p		Aniseed	**Hau_Tuphoxin1**	*Halocynthia aurantium*
Haaura.CG.MTP2014.S1209.g07864.01.p		Aniseed	H.aurantium.1	*Halocynthia aurantium*
Phfumi.CG.MTP2014.S5765.g07096.01.p	b)	Aniseed	Ph.mammillata.1	*Phallusia mammillata*
Phfumi.CG.MTP2014.S5765.g07097.01.p	b)	Aniseed	Ph.mammillata.2	*Phallusia mammillata*
CAB3267405.1		GenBank	Ph.mammillata.3	*Phallusia mammillata*
Moocul.CG.ELv1_2.S110138.g11514.01.p		Aniseed	M.oculata.1	*Molgula oculata*
Moocul.CG.ELv1_2.S61057.g03850.01.p		Aniseed	M.oculata.2	*Molgula oculata*
Moocci.CG.ELv1_2.S255256.g05916.01.p		Aniseed	M.occidentalis.1	*Molgula occidentalis*
Moocci.CG.ELv1_2.S565922.g22193.01.p		Aniseed	M.occidentalis.2	*Molgula occidentalis*
Moocci.CG.ELv1_2.S454880.g14754.01.p		Aniseed	M.occidentalis.3	*Molgula occidentalis*
Moocci.CG.ELv1_2.S568724.g22364.01.p		Aniseed	M.occidentalis.4	*Molgula occidentalis*
CJ410700.1		GenBank	M.tectiformis	*Molgula tectiformis*
XP_026691523.1		GenBank	C.intestinalis.2	*Ciona intestinalis*
XP_002122867.2		GenBank	C.intestinalis.1	*Ciona intestinalis*
XP_018672468.2		GenBank	C.intestinalis.3	*Ciona intestinalis*
XP_018671739.2		GenBank	C.intestinalis.4	*Ciona intestinalis*
XP_002119675.4		GenBank	C.intestinalis.5	*Ciona intestinalis*
XP_002128425.2		GenBank	C.intestinalis.6	*Ciona intestinalis*
Boleac.CG.SB_v3.S604.g12287.01.p		Aniseed	B.leachii.1	*Botrylloides leachii*
Boleac.CG.SB_v3.S604.g12288.01.p		Aniseed	B.leachii.2	*Botrylloides leachii*
Boleac.CG.SB_v3.S227.g05332.01.p		Aniseed	B.leachii.3	*Botrylloides leachii*
Boleac.CG.SB_v3.S227.g05333.01.p		Aniseed	B.leachii.4	*Botrylloides leachii*
Boleac.CG.SB_v3.S227.g05334.01.p		Aniseed	B.leachii.5	*Botrylloides leachii*
Boleac.CG.SB_v3.S227.g05338.01.p		Aniseed	B.leachii.6	*Botrylloides leachii*
Boleac.CG.SB_v3.S195.g04406.01.p		Aniseed	B.leachii.7	*Botrylloides leachii*
Boleac.CG.SB_v3.S281.g06648.01.p		Aniseed	B.leachii.8	*Botrylloides leachii*
JG332329.1		GenBank	B.schlosseri.1	*Botryllus schlosseri*
JG338731.1		GenBank	B.schlosseri.2	*Botryllus schlosseri*
AWK28007.2		GenBank	P.prolifica.1	*Phyllochaetopterus prolifica*
AWK28006.2		GenBank	P.prolifica.2	*Phyllochaetopterus prolifica*

AWK28003.1	GenBank	M. taylori	*Mesochaetopterus taylori*
EY523895.1	GenBank	C. teleta	*Capitella teleta*
CAQ64614.1	GenBank	T. chilensis	*Tonicia chilensis*
CAQ64609.1	GenBank	N. mirandus	*Nuttallochiton mirandus*
CAQ64607.1	GenBank	N. conica	*Notoplax conica*
CAQ64615.1	GenBank	A.fascicularis	*Acanthochitona fascicularis*
CAQ64608.1	GenBank	N.fluxa	*Nuttallina fluxa*
CAQ64600.1	GenBank	C.magnificus	*Chiton magnificus*
CAQ64603.1	GenBank	L.radians	*Lepidozona radians*
CAQ30429.1	GenBank	C.bouveti	*Callochiton bouveti*
CAQ30427.1	GenBank	N.lineata	*Nierstraszella lineata*
CAQ64612.1	GenBank	S.brandtii	*Schizoplax brandtii*
AYO86686.1	GenBank	H.pomatia.1	*Helix pomatia*
AYO86688.1	GenBank	H.pomatia.2	*Helix pomatia*
AEO51768.1	GenBank	H.lucorum	*Helix lucorum*
AYO86691.1	GenBank	L.stagnalis	*Lymnaea stagnalis*
CEK95569.1	GenBank	A.vulgaris.1	*Arion vulgaris*
CEK81230.1	GenBank	A.vulgaris.2	*Arion vulgaris*
CEK79687.1	GenBank	A.vulgaris.3	*Arion vulgaris*
CEK74026.1	GenBank	A.vulgaris.4	*Arion vulgaris*
CEK95583.1	GenBank	A.vulgaris.5	*Arion vulgaris*
CEK74025.1	GenBank	A.vulgaris.6	*Arion vulgaris*
AYO86690.1	GenBank	A.vulgaris.7	*Arion vulgaris*
AYO86689.1	GenBank	A.vulgaris.8	*Arion vulgaris*
DW255933.1	GenBank	E.scolopes.1	*Euprymna scolopes*
DW256698.1	GenBank	E.scolopes.2	*Euprymna scolopes*
DAC71535.1	GenBank	A.californica.1	*Aplysia californica*
CAD88977.1	GenBank	A.californica.2	*Aplysia californica*
XP_035826072.1	GenBank	A.californica.3	*Aplysia californica*
AYO86685.1	GenBank	C.aspersum	*Cornu aspersum*
PVD31865.1	GenBank	P.canaliculata.1	*Pomacea canaliculata*
PVD32161.1	GenBank	P.canaliculata.2	*Pomacea canaliculata*
PVD32038.1	GenBank	P.canaliculata.3	*Pomacea canaliculata*
CAH10287.1	GenBank	N.nucleus.1	*Nucula nucleus*
CAH10286.1	GenBank	N.nucleus.2	*Nucula nucleus*
CAG28308.1	GenBank	M.crenulata.1	*Megathura crenulata*
4BED_B	GenBank	M.crenulata.2	*Megathura crenulata*
CAC82192.1	GenBank	H.tuberculata	*Haliotis tuberculata*
ANE23704.1	GenBank	H.rubra.1	*Haliotis rubra*
CX726143.1	GenBank	H.discus	*Haliotis discus*
GT869640.1	GenBank	H.diversicolor	*Haliotis diversicolor*
ABD47516.1	GenBank	S.officinalis.1	*Sepia officinalis*
CBW46988.1	GenBank	L.cinerea	*Lepidochitona cinerea*
AGX25261.1	GenBank	M.tuberculata	*Melanoides tuberculata*
O61363.1	GenBank	E.dofleini.1	*Enteroctopus dofleini*
AAA29391.1	GenBank	E.dofleini.2	*Enteroctopus dofleini*
4YD9_A	GenBank	T.pacificus.1	*Todarodes pacificus*
4YD9_B	GenBank	T.pacificus.2	*Todarodes pacificus*
ADT91166.1	GenBank	S.spirula	*Spirula spirula*
AGV74427.1	GenBank	S.maindroni	*Sepiella maindroni*

CAF03590.1	GenBank	N.pompilius	*Nautilus pompilius*
XP_014789836.1	GenBank	O.bimaculoides.1	*Octopus bimaculoides*
XP_014779647.1	GenBank	O.bimaculoides.2	*Octopus bimaculoides*
XP_029636914.2	GenBank	O.sinensis	*Octopus sinensis*
HX972596.1	GenBank	P.acuta	*Physella acuta*
ES748875.1	GenBank	B.glabrata	*Biomphalaria glabrata*
JK740481.1	GenBank	M.acridum	*Metarhizium acridum*
FP450128.1	GenBank	T.melanosporum	*Tuber melanosporum*
FG247591.1	GenBank	C.zeae-maydis	*Cercospora zeae-maydis*
CO033831.1	GenBank	C.posadasii	*Coccidioides posadasii*
EX805973.1	GenBank	L.bicolor	*Laccaria bicolor*
WP_088241413.1	GenBank	C.rhizosoleniae	*Calothrix rhizosoleniae*

Footnotes:

a) - overlaping sequences, merged to give Tuphoxin transcript of Styela rustica

b) - overlaping sequences, merged under the name of Ph.mammillata.1.2

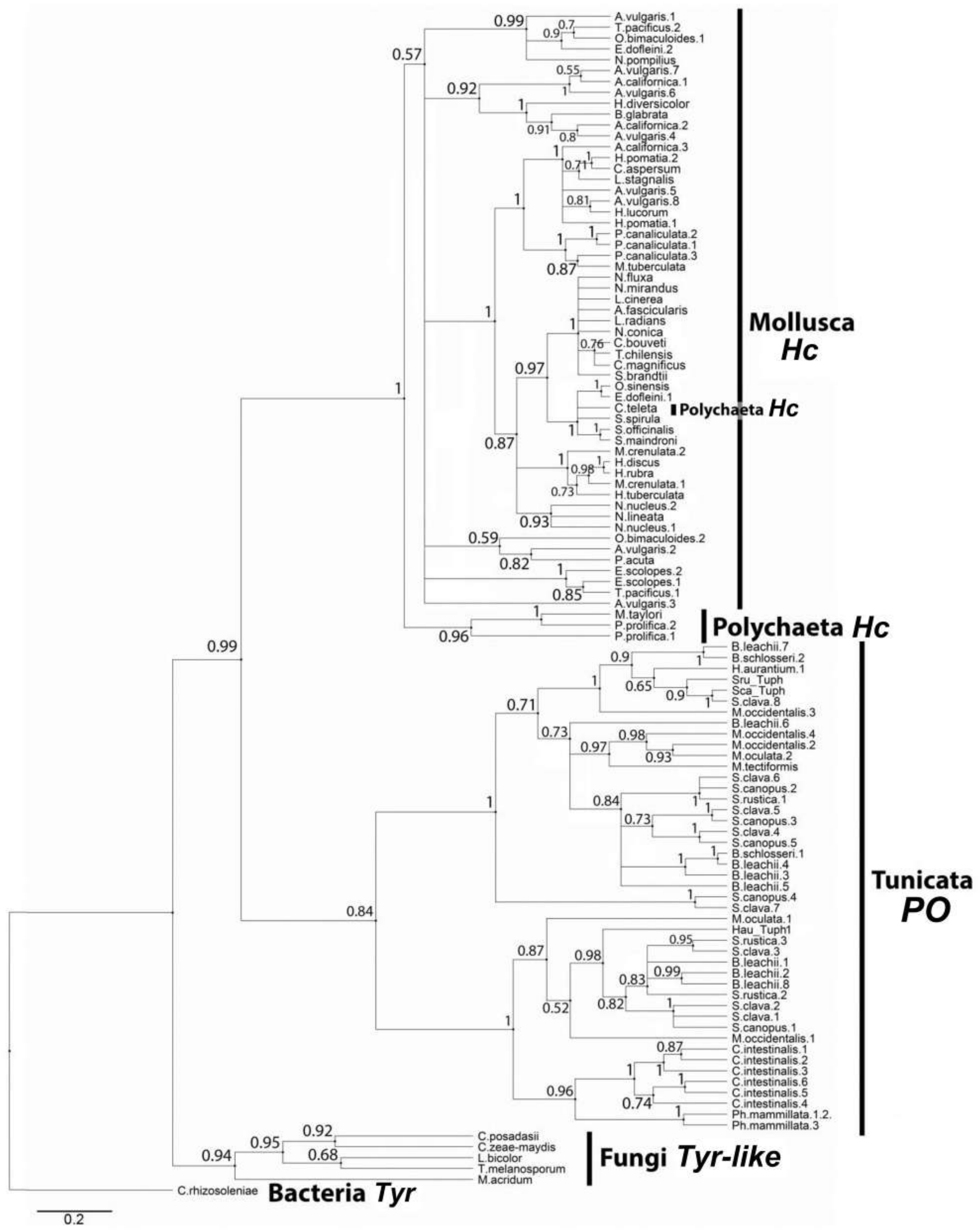

Supplementary Figure S9. Phylogenetic analysis of tyrosinase domains of *Tuphoxin* homologues. Phylogenetic tree based on Bayesian Inference (BI) rooted by bacterial sequence. Statistical support (posterior probabilities) are indicated at the nodes. Only posterior probabilities >0.5 are shown. Accession numbers of the proteins used in this tree can be found in Supplementary Table S1.

Supplementary Table S2 Arthropod-like phenol oxidases of ascidians used for phylogeny construction.

Database ID	Sequence name	Species	Protein name	Citation
XP_018671030.1	C.intestinalis.7	*Ciona intestinalis*	tyr1	Aguilera et al., 2013
NP_001029009.2	C.intestinalis.8	*Ciona intestinalis*	tyr3	Aguilera et al., 2013
CAD68058.1	C.intestinalis.9	*Ciona intestinalis*	tyr4	Aguilera et al., 2013
AJ547814	C.intestinalis.10	*Ciona intestinalis*		Immesberger, Burmester, 2004
ALM04212.1	B.schlosseri.3	*Botryllus schlosseri*	HLP	Ballarin et al., 2012

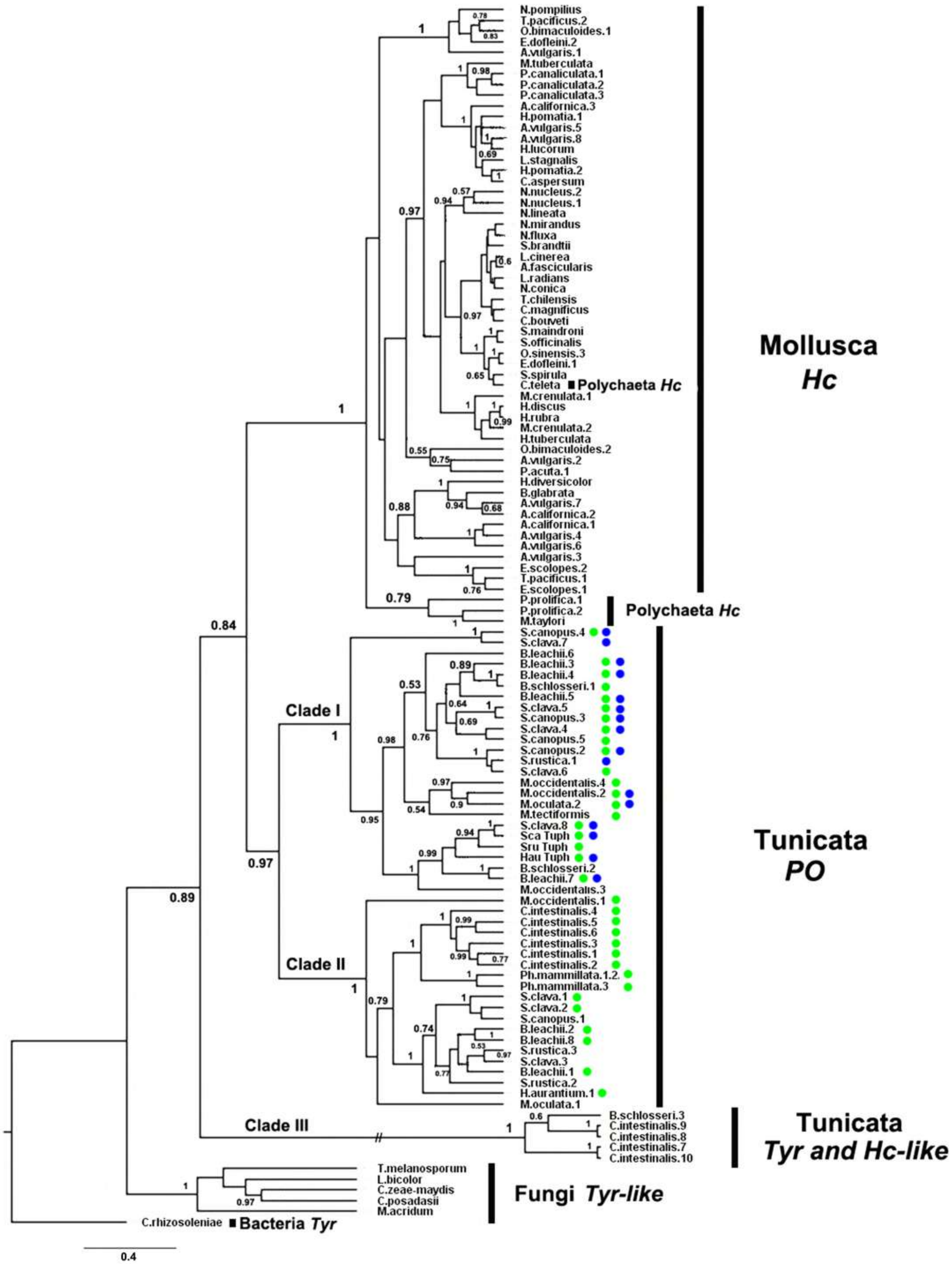

Supplementary Figure S10. Phylogenetic analysis of tyrosinase domains of *Tuphoxin* homologues and tyrosinase domains arthropod-like PO of ascidians. Phylogenetic tree based on Bayesian Inference (BI) rooted by bacterial sequence. Posterior probabilities >0.5 are shown in nodes. Hc – haemocyanin, PO – phenol oxidase, Tyr – tyrosinase. Accession numbers of the sequences used in this tree can be found in Supplementary Table S1 and S2.

Supplementary Table S3. Number of unique alleles of *Tuphoxin* homologues present in Tunicata. Eleven ascidian species discussed in the present study are listed with number of alleles in each of two clades of Tunicata homologues. Clades are supported by maximum likelihood (ML) inference of phylogenetic relations.

	**number of unique allels**	
**species**	**clade I**	**clade II**
Styela rustica	2	2
Styela canopus	5	1
Styela clava	5	3
Halocynthia aurantium	1	1
Phallusia mammillata	0	1
Molgula oculata	1	1
Molgula occidentalis	3	1
Molgula tectiformis	1	0
Ciona intestinalis	0	6
Botryllus schlosseri	2	0
Botrylloides leachii	5	3

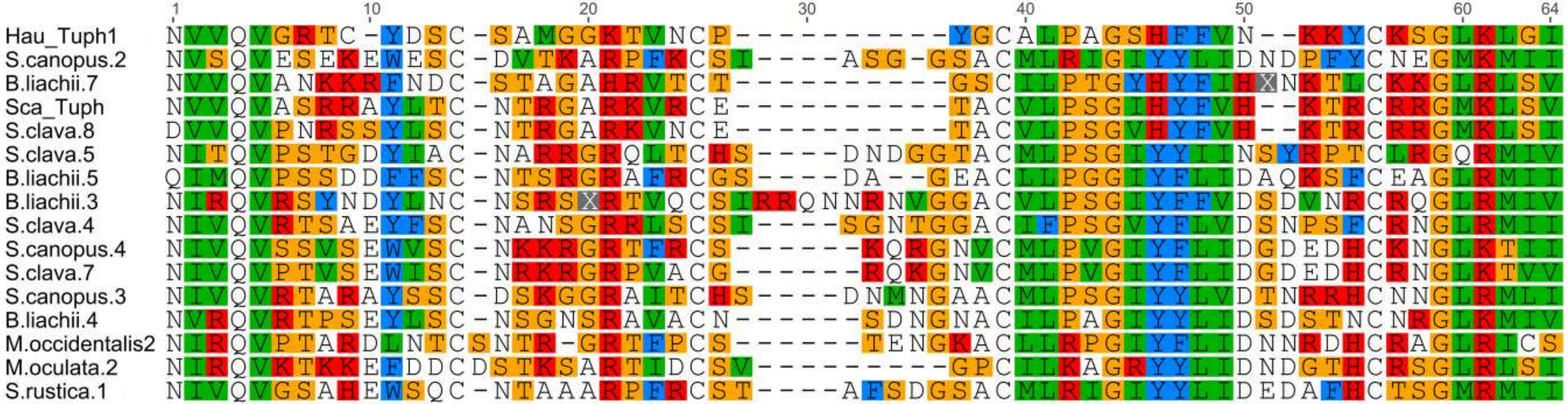

Supplementary Figure S11. Alignment of cupedoxin-like sequences of *Tuphoxins* and their homologues among Tunicata.

Sca_*Tuph* – *Tuphoxin* transcript belonging to *S. canopus*, Hau_*Tuph1* – *Tuphoxin* transcript belonging to *H. aurantium.*

Supplementary Table S4. Presence or absence of TSP1 domain and/or cupredoxin-like sequence (CuOx-like) in full length homologues of *Tuphoxin* belonging to Tunicata.

Database ID	Name	Species	TSP1	CuOx-like
Phfumi.CG.MTP2014.S5765.g07096.01.p	Ph.mammillata.1.2	*Phallusia mammillata*		
CAB3267405.1	Ph.mammillata.3	*Phallusia mammillata*		
Moocul.CG.ELv1_2.S110138.g11514.01.p	M.oculata.1	*Molgula oculata*		
Moocul.CG.ELv1_2.S61057.g03850.01.p	M.oculata.2	*Molgula oculata*		
Moocci.CG.ELv1_2.S255256.g05916.01.p	M.occidentalis1	*Molgula occidentalis*		
Moocci.CG.ELv1_2.S565922.g22193.01.p	M.occidentalis2	*Molgula occidentalis*		
Moocci.CG.ELv1_2.S454880.g14754.01.p	M.occidentalis3	*Molgula occidentalis*		
Moocci.CG.ELv1_2.S568724.g22364.01.p	M.occidentalis4	*Molgula occidentalis*		
CJ410700.1.p3	M.tectiformis	*Molgula tectiformis*		
XP_002122867.2	C.intestinalis.1	*Ciona intestinalis*		
XP_026691523.1	C.intestinalis.2	*Ciona intestinalis*		
XP_018672468.2	C.intestinalis.3	*Ciona intestinalis*		
XP_018671739.2	C.intestinalis.4	*Ciona intestinalis*		
XP_002119675.4	C.intestinalis.5	*Ciona intestinalis*		
XP_002128425.2	C.intestinalis.6	*Ciona intestinalis*		
Boleac.CG.SB_v3.S604.g12287.01.p	B.liachii.1	*Botrylloides leachii*		
Boleac.CG.SB_v3.S604.g12288.01.p	B.liachii.2	*Botrylloides leachii*		
Boleac.CG.SB_v3.S227.g05332.01.p	B.liachii.3	*Botrylloides leachii*		
Boleac.CG.SB_v3.S227.g05333.01.p	B.liachii.4	*Botrylloides leachii*		
Boleac.CG.SB_v3.S227.g05334.01.p	B.liachii.5	*Botrylloides leachii*		
Boleac.CG.SB_v3.S227.g05338.01.p	B.liachii.6	*Botrylloides leachii*		
Boleac.CG.SB_v3.S195.g04406.01.p	B.liachii.7	*Botrylloides leachii*		
Boleac.CG.SB_v3.S281.g06648.01.p	B.liachii.8	*Botrylloides leachii*		
AA49_m.7122.p1	S.clava.1	*Styela clava*		
AA49_m.7119.p1	S.clava.2	*Styela clava*		
AA49_m.30831.p1	S.clava.3	*Styela clava*		
AA49_m.87151.p1	S.clava.4	*Styela clava*		
AA49_m.2942.p1	S.clava.5	*Styela clava*		
AA49_m.30764.p1	S.clava.6	*Styela clava*		
AA49_m.72475.p1	S.clava.7	*Styela clava*		
AA49_m.3326.p1	S.clava.8	*Styela clava*		
SC_m.117002.p1	**Sca_Tuphoxin**	*Styela canopus*		
SC_m.152480.p1	S.canopus.1	*Styela canopus*		
SC_m.16402.p1	S.canopus.2	*Styela canopus*		
SC_m.86302.p1	S.canopus.3	*Styela canopus*		
SC_m.42250.p1	S.canopus.4	*Styela canopus*		
SC_m.26137.p1	S.canopus.5	*Styela canopus*		
Contig_28777+NODE_67444	**Sru_Tuphoxin**	*Styela rustica*		
Contig_7091	S.rustica.1	*Styela rustica*		
Contig_7704	S.rustica.2	*Styela rustica*		
Contig_15578	S.rustica.3	*Styela rustica*		
Haaura.CG.MTP2014.S1292.g08050.02	**Hau_Tuphoxin1**	*H.aurantium*		
Haaura.CG.MTP2014.S1209.g07864.01	H.aurantium.1	*H.aurantium*		
JG332329.1.p1	B.schlosseri.1	*Botryllus schlosseri*		
JG338731.1.p1	B.schlosseri.2	*Botryllus schlosseri*		

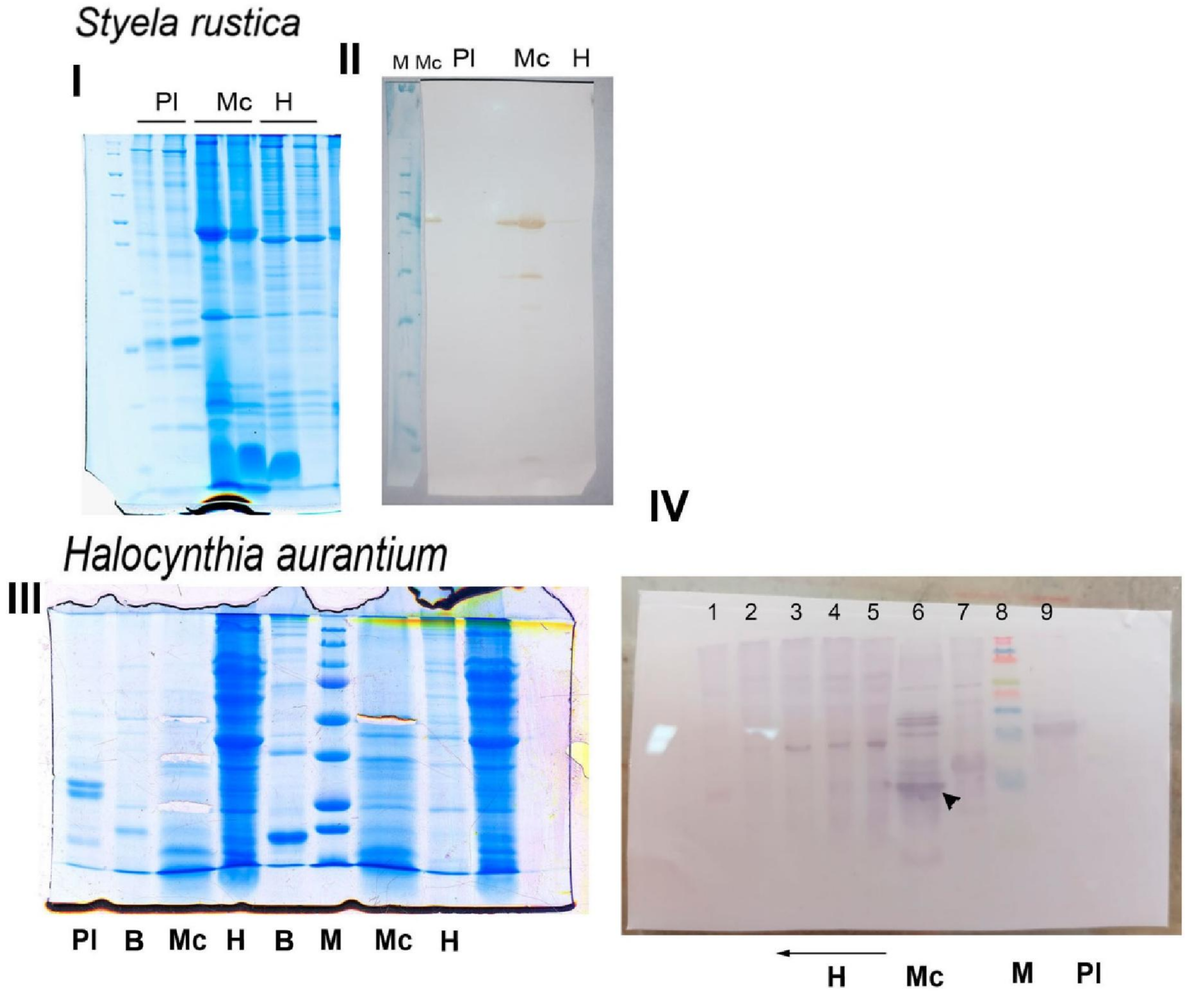

Supplementary Figure S12. Examples of the original electrophoresis and immunoblot of the blood cells from the species indicated. I, III - SDS electrophoresis; II, IV - immunoblot. Hyalinocytes (H), morula cells (Mc), blood plasma (Pl), bottom fraction of degranulated cells (B) and marker proteins ladder (M) At III the lines visible cut out for MALDI. At IV arrow at the bottom indicate the decreasing loading of hyalinocyte fraction; arrowhead indicate the false positive signal belongs to the debris. The polished image aligned according to the markers is shown at Figure 2 in the text.

```
>Sru_Tuph_cloned cDNA
CGTATCGCGATGCATCTAGATTGCACTGCGAGGTTGTCATACCTGTATCCCAGCAATTCCCTGTAGTAAAACATATTCTGGATGCTGGCGAGTTCCTGAAGCTTTTTGAATGGAGTGAGAG
TCTTGTTTCTGAACGGCAGGTATGGTTCATTGAAATGGAAGATGTTTTCTTGAGGTTCAAGCACAGACTGTCTGGTCCAAGCTTCTTCGCCATGAGCTGACATCCAGTTTTGGAAAACGGA
GAAAAGTCTGTCGACGTTTGCATGATGAAAAAAGAAGACTGGATCGAATCCCGCATATGTGAGACTACCCATTGAGTAGGTACATTTGCCAGCGGCTGATTCCTTTCCCCAGCAAATAGCA
GCACGAATGGTGTTGTGAGAGTTCTCAAGAGCCTCGGAAAACTCATGGATGGTGTGTGACTGCATCAACTTTTGTGCAACATAGTAGTTGTCCTTCGACACTTTGACCATGAAAGGATAGA
CCGTGGGGGAACGTTGTGTCAGGGTATGCGGGGTAGTGTCGTATCGAATAGTCATGGACGTAAACGGATTTTCCTTGCCGTATAGTTTCTTGCTCCAAACCAATGCAGGCGGCTCAGGATA
TTTAAGCCAGTCCCAGTAAGGCAATCCTAGAGTTTTATCTTTGAGATGCCTTGTAAGTCCTTCTTCAAAGTTCAATAATTCAGCTCTGTGCCACATTGCAAAGTTGAGTCTTGCACCATGG
TAGCAACATCCTGCCATATTTCCATCTGGTCCCATGCACTGATAAGGAAAGACGTGAGTCCCAGCCGCATTGTGGAATCCAAAATTGCTAGTATCCTTTTTGTATTTCCTCATCGCTTGAA
TAAGATCTTTCTTCTGTACTCGGTTCAGTGAATGAGCCTCTCTCCTGACACGCAATTTGCATTTTCTCCCGGTGTAGCATTCCTTGTATTCAGTTGCTGGTCCTTTACACCTGCCTTCTCC
CACAAGGCCATGTTTGCAAATACGAGTCCGGGTCTGAATTCCACCTGAACAGGTTGGTTTGCATGTGTTCCAACAGCTCCATGTTTCCCATTTAGCTTCGGTATCAAAGACGTTCTTTACC
GTCCACTTTAGGCCAACGCAGACATAATTGATGATGATGTCAGTTTTGCCATCAACATCTACATCAAACCTTTGCACTCTCTTTGCTCCTCCGGTCTTGCAGGTTTTATACATGAGTGTAT
GAAAA
```

Supplementary data 1. A sequence of *S.rustica* tuphoxin cloned fragment.

```
M.occidentalis.   --MNDTGTHEWPYQCRS-----STGINGACCYHGAKLNFLSWHRVEIKNFEMGLTRYL-----------------KDKTLGMPY
B.leachii.7       GFWAAGGTHVFPNQCVD----HVSGNPGPCCYHGARLNFAIWHRAELLNIEEGITRYM-----------------KDKTLGLPY
B.schlosseri.2    GFWAAAGTHVFPNQCID----HATGNPGPCCYHGARLNFAIWHRAELLNIEEGLIRHL-----------------KSKTLGLPY
Hau               GYHQAAGTHFWPNECVK-----PNGVSGACCYHGAKVNFVMWHRAELLYYEQGLTRHL-----------------KDKKLGAPY
Sca               GFHNAGGTHEFPFQCMG-----SSGEDKGCCYHGARLNFVMWHRAELLNFEEGLSRHL-----------------RDKTLGLPY
S.clava.8         GFHNAGGTHEFPFQCMG-----SDGKDKGCCFHGAKLNFAMWHRAELLNFEEGLTRHL-----------------KDKTLGLPY
M.acridum         GYFQISGIHGLPFIEWNRGGARNNDGWGGYCPHGEAL-FLPWHRPFVLLFEQLLVEHAVGIASQYPARYRGQYMAAANNLRSPY
T.melanosporum    GYYQVAGVHGTPYIPWMEKA-DADDRAGDYCTHGTAL-FLTWHRPYLLLFEQRIVEEALTIARNFSDKYRAEYEEAALNIRIPY
C.zeae-maydis     -VQKCADCQGA----------------DGYCTHDSVH-FPAWHRPYMALFEQELMRVAFDIASSFSGAFGDRLKAAAQKLRAPY
C.posadasii       SYYDVSSIHGYPFEAWDG---VEGIGGGGYCAHASTI-FPTWHRPYLALFEEMIWNHAQVIARGYRDSLRSTYVEAARTLRIPY
C.rhizosoleniae   GYEQIAGLHGFPDW---------------ECPHGGLK-FLPWHRAYLLRFEQALQRYV-------------------PDVTLPY
L.bicolor         SFFQVGGIHGLPYIPWDGVTGIPNPTWGGYCTHGSVL-FPTWHRPYVSAFEQILWTRAQEVAATYTVD-QAAWKAAATTLRQPY
BLOCKs            SFFQVGGIHGLPYIPWD------------YCTHGF---FPTWHRPYVSAFEQI-----------------------ATTLRQPY

cons                    . :                     * *     *  ***  :   *                            :  **


M.occidentalis.   WNWLE-Y----SS---PPPLLW-SKTL-----------------YGATNPFAEPMEVRLDE------------------SNIRK
B.leachii.7       WNWLK-E----NK---APDFVW-SKTL-----------------NGKPNPLAH-IIIRHDL------------------TPPQP
B.schlosseri.2    WNWLK-D----NK---APDFVW-SKTY-----------------NGKPNPFAN-VIIRYDL------------------TPHQA
Hau               WDWLV-Y----PN---PPQLVW-AKTI-----------------GGKPNPFAG-SKIRYDL------------------PVVIS
Sca               WDWLT-Y----PN---PPPLVW-SKKL-----------------YNKANPFTS-GLVRYDT------------------KPHTL
S.clava.8         WDWLT-Y----PN---PPPLVW-SKKL-----------------FKKDNPFTS-MTIRYDV------------------TPNIV
M.acridum         WDWSADS----NV---PPCTVP-ERVS-VNIP-----NGQNLRRIEIRNPLST-YKYPREA---LDGEF---GEFSKTPQMARC
T.melanosporum    WDWATDSD--------VPQSIR-FAETDITLPEVGSDAPPV-TRKGVPNPMYS-YKFKTSIRRQRDFSIVGVQEMVAWEETKRC
C.zeae-maydis     WDWAAVPDPGTPV---LPTVMT-AQQVTI--------EGPN-GQETVDNPLYS-YHFNDSS----DMAY---AVFTTWPDTFRW
C.posadasii       WDWASNP-----E---LPRCVV-TPQIRI--------TTPN-GEATIKNPLYS-YTINPTA----GKGFPPRDPLSQYPDTVRA
C.rhizosoleniae   WDWISQE----SQRNGLPNAVA-ESTY----------FDA--EGVEVLNPLYS-AQIPIGG------------------ETIRR
L.bicolor         WDWAVNS-----V---PPDEVIAKQTVSI--------TGHDGKKKDVANPLYH-YTFHPI-----DPSFP--SPYSGWKTTLRQ
BLOCKs            WDWA------------PPDEV---------------------------------------------------------------

cons              *:*              *  :
```

```
M.occidentalis.   T---------ERIAQPVP-AHVEVARGIYQNETVNL-QSADMIHEFSES-------------LESSHNLIHMAVC-PMTPNSKA
B.leachii.7       L---------QRDNTTQL-SSILQARSIYLTTAMKL-MESHTLHEFSEE-------------LENAHNWIHTAAC-PY--ETPA
B.schlosseri.2    V---------QRDTTTDF-NFIKLARDIYLTTTIRL-MKAHSLFRFSEE-------------LENAHNWIHSSAC-PH--ETSA
Hau               T---------ARSPAVSS-SSVSMAKSIFQTITSKL-ARSHMIHEYSES-------------LESSHNNIHSAVC----HADTG
Sca               T---------QRSPTVDP-FMVKMSKDNY-YVARKL-MQSHSIHEFSEA-------------LENSHNTIHSSICWGAQQKAGK
S.clava.8         T---------QRSPTVDP-FMVKTSKDNY-YVARKM-MQSHSIHEFSEA-------------LENAHNSIHSSICWGAQQNQNK
M.acridum         PDPG--RYPDSANQELQD--------YRLKQATYDIFTTASNFHQFAVRG--------RVHHLEELHNDVHYSAA---------
T.melanosporum    PDEKGISHPEIVDRQLRIPTVNPTAGSSFRDPIYKLLTLVGSYGAFGNTGWQTGRPGPNNISLEHYHNIIHTFTG-TNYIEEKF
C.zeae-maydis     PNSNNVDAHSQQDQCID---AFTNSNGNLRDQIYQLLTQCKSYLGFATDA--SGDQ-RCANSLEGIHNTVHLNAG-GPGY-NGI
C.posadasii       PDDEGQSQMEEVQRRLRA------NGAYFRQATYSLLSSEPNYTIFSTSA--VRDRGNNYNNLETIHGQVHVAVG-GPG-----
C.rhizosoleniae   P------------------GQISRLSELENPVAIAM--QENNFVDFSGR-------------IENPHNFIHVWVG-GNG-----
L.bicolor         PTSLQPD---ATDNIVRLRQVLGNAQSNIRSNTYNLLTRVTTWPAFSNHT--VGDGGSTSNSLEAIHDGIHVYVG-GNG-----
BLOCKs            --------------------------------------RVTTWPAFSNH--------------EAIHDGIHVYVG---------

cons                                                           :.                *  *. :*


M.occidentalis.   CEVSMGSLTYAAFDPTFMFHHTNVDRLFSQF
B.leachii.7       CTYSMGQLTYAAYDPLFFMHHTNVDRQIARL
B.schlosseri.2    CTFSMGQLEYAAYDPLFFMHHTNVDRQIARM
Hau               CDYSMGELEYAAYDPIFFFHHANVDRLFSVF
Sca               CEFSMGGLTYAAFDPVFFFHHTNVDRLFSVF
S.clava.8         CTYSMAGLTYAAFDPVFFFHHTNIDRLFSVF
M.acridum         CKGQFFDAELSGFEPLFMFHHTQVDRLW---
T.melanosporum    QEGHMSEVGVSAFDPIFWLHHCNVD------
C.zeae-maydis     SGGHMAFLPLASYDPLFFLHHANVDRLVTLY
C.posadasii       --GHMTYVPWSSFDPVFWLHHANVDRLLAMW
C.rhizosoleniae   --GSMSNVTFAAFDPIFWMHHSYVDRQWYIW
L.bicolor         ---QMGDPAVAGFDPIFFLHHCNVDRLLSLW
BLOCKs            --GQMGDPAVAGFDPIFFLHHCNVDR-----

cons                  :     :.::* * :**  :*
```

Supplementary data 2. Alingment of Tunicate and fungal phenol oxidase proteins. BLOCKs indicate the most conservative regions. Color meaning for alignment: BAD AVG GOOD
